# Norbornene chaotropic salts as low molecular mass ionic organogelators (LMIOGs)†
†Electronic supplementary information (ESI) available: Synthesis, NMR diffusion, gelation, SEM images, DSC thermograms, rheological data and crystallographic data. CCDC 1826288–1826290. For ESI and crystallographic data in CIF or other electronic format see DOI: 10.1039/c8sc01798k


**DOI:** 10.1039/c8sc01798k

**Published:** 2018-05-16

**Authors:** Jordan R. Engstrom, Aramballi J. Savyasachi, Marzieh Parhizkar, Alessandra Sutti, Chris S. Hawes, Jonathan M. White, Thorfinnur Gunnlaugsson, Frederick M. Pfeffer

**Affiliations:** a School of Life and Environmental Sciences , Deakin University , Waurn Ponds , Victoria 3216 , Australia . Email: fred.pfeffer@deakin.edu.au; b School of Chemistry and Trinity Biomedical Sciences Institute (TBSI) , Trinity College Dublin , The University of Dublin , Dublin 2 , Ireland . Email: gunnlaut@tcd.ie; c Institute for Frontier Materials , Deakin University , Waurn Ponds , Victoria 3216 , Australia . Email: asutti@deakin.edu.au; d School of Chemical and Physical Sciences , Keele University , Staffordshire , ST5 5BG , UK; e Bio21 Institute , School of Chemistry , University of Melbourne , Parkville , 3010 , Australia

## Abstract

Phenylalanine functionalised norbornene (**9:Na**) functions as a potent, low molecular-mass (*M*_W_ = 333 Da) ionic organogelator with a minimum gelating concentration of 0.5 wt% in THF, *i*-PrOH, 1,4-dioxane and *n*-BuOH. Fibrous crystals form in the gel and X-ray crystallography identified a cation mediated helical assembly process controlled by the chirality of the phenylalanine. In addition to excellent gelating properties **9:Na** readily forms aqueous biphasic and triphasic systems.

## Abstract 2

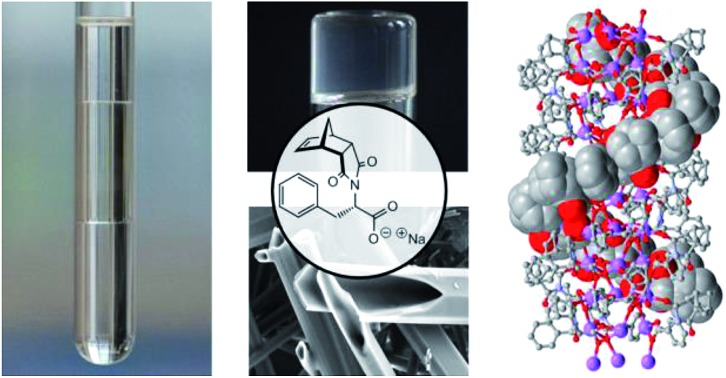
A humble norbornene functions as an ionic organogelator, forms aqueous biphasic and triphasic systems and assembles to form chiral helices.

## Introduction

The development of efficient organogelating agents remains an important pursuit as the corresponding organogels have found use in sensing,^1^–^3^ cosmetics,^4^,^5^ water purification^6^,^7^ and drug delivery applications.^8^–^12^ Low molecular mass organogelators (LMOGs) are of particular interest as these can self-assemble using non-covalent interactions^9^,^13^–^16^ to give rise to novel soft materials with a range of entangled and solvent dependent morphologies.^13^,^17^,^18^ Recently we have developed various examples of LMOGs from simple organic ligands that can, through self-association, or *via* coordination to either d- or f-metal ions, give rise to functional self-assembled gels.^19^–^25^ In parallel, our ongoing interest in applications of functionalised norbornanes and related norbornylogous systems,^26^–^33^ led us to synthesise simple norbornanes modified with amino-acids and here we report the development of such functionalised norbornenes as new low molecular mass ionic organogelators (LMIOGs) (*M*_W_ < 350 Da).

**Fig. 1:**
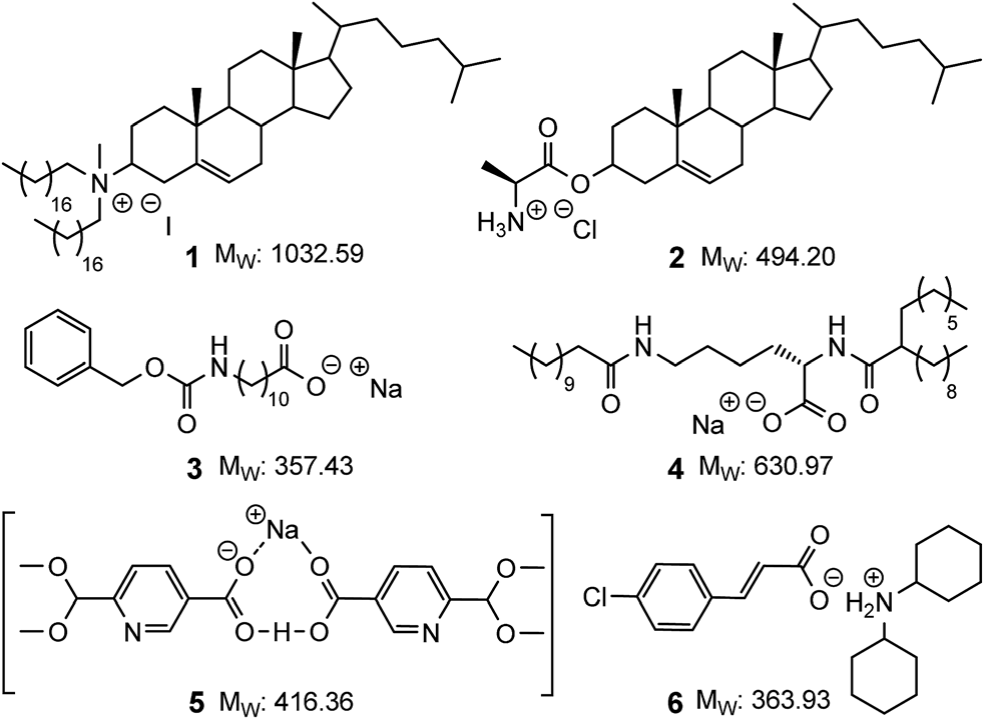
Examples of low molecular mass ionic organogelators. Compounds **1–4** with (and compounds **5** and **6** without) large hydrophobic groups.

Ionic compounds capable of gelating organic solvents are rare and the examples that do exist are typically either based on steroidal frameworks or possess long alkyl chains that give rise to *M*_W_ ranging from ≈350 up to 1000 Da (*e.g.***1–4,**Fig. 1).^9^,^13^,^17^,^34^–^40^ Reports of compact LMIOGs, in which large hydrophobic groups have not been deliberately incorporated are extremely rare (*e.g.***5** and **6**, Fig. 1).^40^,^41^

Herein, we present phenylalanine norbornene **9**, a highly attractive building block for use in supramolecular self-assembly and as a LMIOG. We demonstrate that both the (*R*) and (*S*) enantiomers participate in a chirality controlled, helical assembly process that has been characterised using X-ray crystallographic analysis, and that as a LMIOG, these form gels with fast healable rheological properties. The corresponding xerogels consist of hollow tubular microcrystalline structures as identified using scanning electron microscopy (SEM).

## Results and discussion

**Scheme 1:**
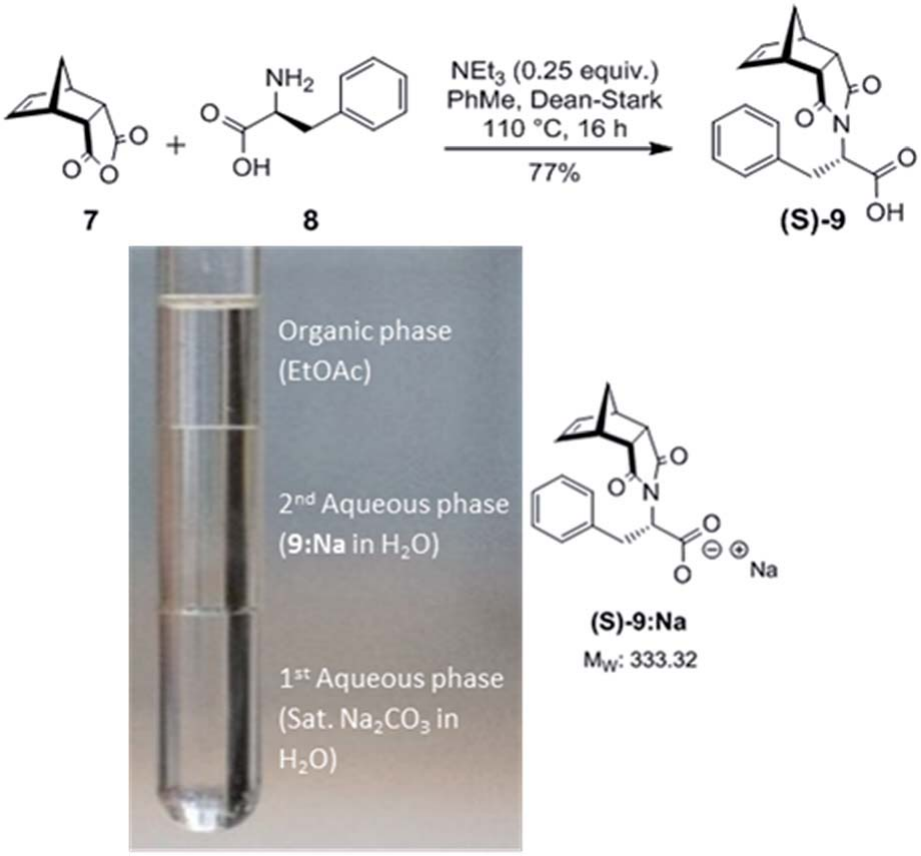
Top: Synthesis of phenylalanine-functionalised norbornene **(*S*)-9**. Bottom: Triphasic system formed by mixing **(*S*)-9** with sat. Na_2_CO_3_ and EtOAc.

During the synthesis of (*S*)-phenylalanine-functionalised norbornene **(*S*)-9** (Scheme 1) from readily available precursors, a triphasic system formed upon workup using aqueous Na_2_CO_3_. While there are a number of literature reports describing compound **9**, no mention of this very interesting phenomenon has been noted.^42^–^46^ The triphasic system (Scheme 1) was characterised as EtOAc (top layer), the added Na_2_CO_3_ (bottom) and the sodium salt of **9** (**9:Na**, middle).

The corresponding aqueous biphasic system could be initiated by simply mixing a concentrated (≥1.8 M) aqueous solution of **9:Na** with sat. Na_2_CO_3_ (see ESI Fig. S1.4†). Aqueous biphasic systems have demonstrated utility in delicate separations such as those involving aliphatic carboxylic acids.^47^–^50^ The biphasic systems also formed when sat. K_2_CO_3_, 5 M NaOH and 5 M KOH solutions were used. As these bases are known kosmotropic salts, the norbornene sodium salt **9:Na**, was suspected of being strongly chaotropic and the two phase system was a result of high concentrations of a kosmotrope and chaotrope.^50^–^52^ To confirm this theory ammonium sulphate (a kosmotropic salt) was added to a concentrated aqueous solution of **9:Na** and the rapid formation of a biphasic system validated these suspicions.

**Fig. 2:**
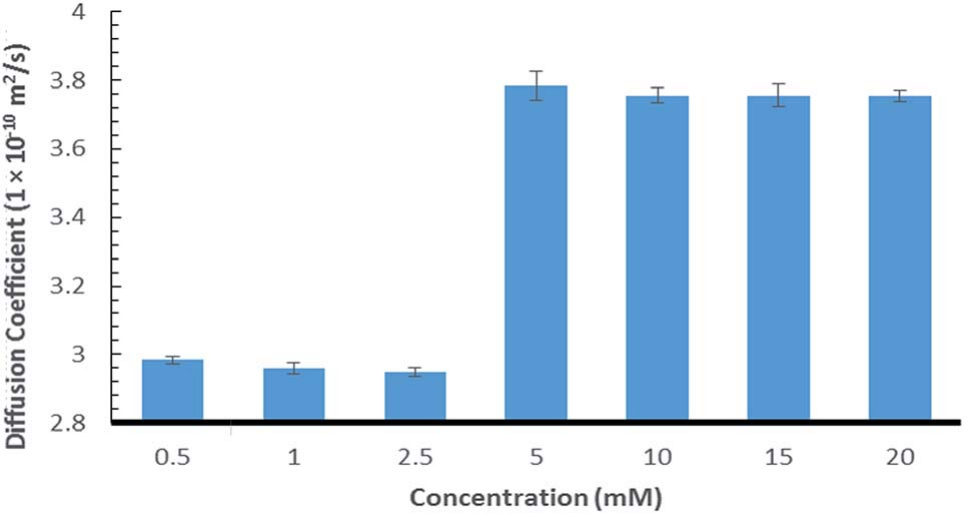
Diffusion coefficient of **9:Na** in water in relation to concentration.

Further evidence of the chaotropic nature was obtained using ^1^H NMR diffusion experiments that showed an increase in the diffusion rate as the concentration of **9:Na** was elevated (Fig. 2, see ESI section S2† for details). This is likely caused by a decrease in the viscosity of the D_2_O, which can in turn, be attributed to the known ability of chaotropic salts to “disrupt” the structure of water.^53^–^55^

### Organogelation

**Fig. 3:**
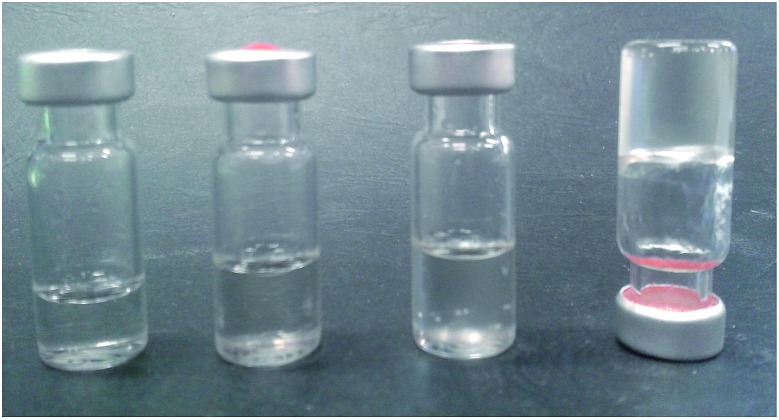
Concentrated aqueous solutions of **9:Na**; from left to right: 1, 2, 2.5 and 3 g mL^–1^.

It was also noted that **9:Na** was exceptionally water soluble, up to 3 g mL^–1^ with heating. Upon cooling, this highly concentrated solution formed a hydrogel (rather than a precipitate) with gel formation likely caused by assembly of a supramolecular polymer.^56^,^57^ Other concentrated solutions (2–2.5 g mL^–1^), did not form gels; instead resulting in the formation of highly viscous solutions, as shown in Fig. 3, again indicating that a larger assembly was likely to be forming under these experimental conditions.

During the characterisation of the biphasic and triphasic systems, it was serendipitously discovered that addition of small amounts (10 μL) of aqueous **9:Na** (≥3 M) to a variety of organic solvents (1.0 mL) resulted in the rapid formation of clear gels. As such, a range of salts of **9** were prepared, including Ca^2+^, K^+^, Na^+^, Li^+^ as well as the tetramethylammonium (TMA), tetraethylammonium (TEA) and tetrabutylammonium (TBA), and their gelating properties were investigated in various protic and aprotic solvents including EtOH, *i*-PrOH, *n*-BuOH, 1,4-dioxane, THF, CHCl_3_ and PhMe.

The calcium salt failed to elicit gel formation in any of the solvents trialled, demonstrating that this salt was not functioning as a LMIOG. However, when small amounts (4 mg) of the potassium salt **9:K** were added to a range of these solvents (200 μL) and heated, on cooling the formation of soft-materials with strong gel-like properties (withstanding the classic inversion test) was observed in both *i*-PrOH and *n*-BuOH, while a weak gel was formed in PhMe (Table 1). These results clearly demonstrate the ability of **9:K** to function as LMIOG in a broad range of solvents.

**Table 1:** Gelation of organic solvents (minimum gelation concentration)

Solvent	**9:K** ^*a*^	**9:Na** ^*a*^
Methanol	D	D
Ethanol	D	G^*a*^ (2 wt%)^*b*^
Isopropanol	TG (2 wt%)^*b*^	G^*a*^ (0.5 wt%)^*b*^ ^,^^*c*^
*n*-Butanol	TG (2 wt%)^*b*^	G^*a*^ (0.5 wt%)^*b*^ ^,^^*c*^
Dioxane	A	G^*b*^ (0.5 wt%)^*c*^
Tetrahydrofuran	I	G^*b*^ (0.5 wt%)^c^
Diethyl ether	I	I
Acetone	A	PG
Ethyl acetate	A	PG
Dimethylformamide	D	D
Dimethyl sulfoxide	D	D
Acetonitrile	I	A
Chloroform	A	WG (1 wt%)^*b*^
Dichloromethane	A	S
Toluene	S	A
Pet. spirits (40–60 °C)	I	I
Heptane	I	I

^*a*^Gelator added as a powder (2 mg mL^–1^).

^*b*^Minimum gelator concentration (MGC established using solid **9:K** or **9:Na**—see ESI section S3 for full details.

^*c*^MGC established using 3.0 M solution of **9:Na**—see ESI for full details. G = clear gel, WG = weak gel, PG = partial gel, S = suspension, D = dissolved, I = insoluble, A = aggregate, TG = turbid gel.

**Fig. 4:**
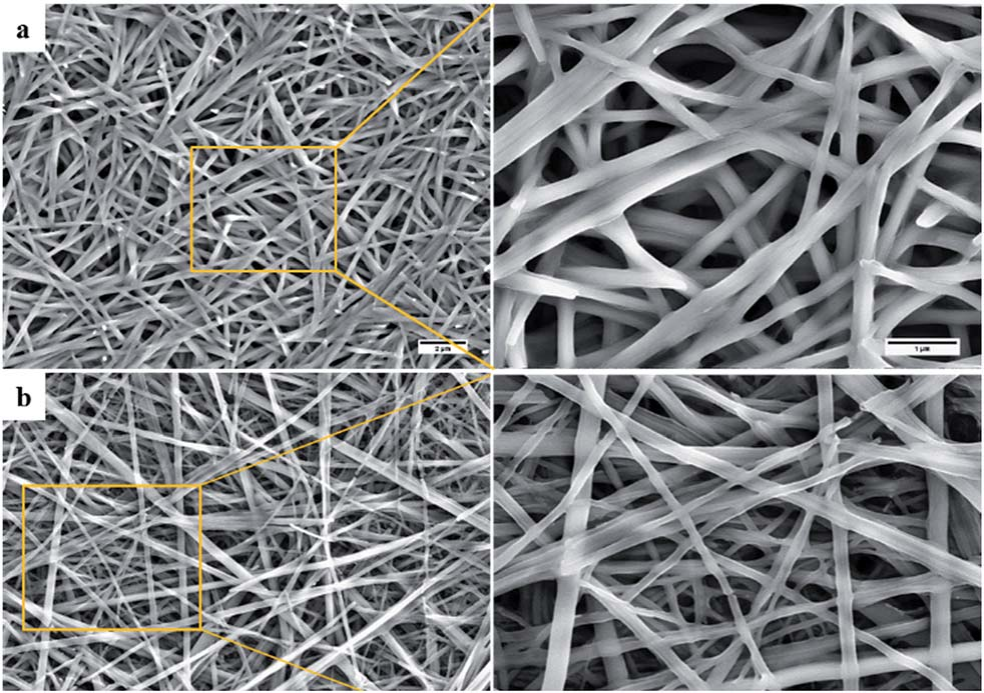
SEM images of **9:K** xerogels obtained from (a) *i*-PrOH gel (2 wt%), (b) *n*-BuOH gel (2 wt%).

We next investigated the morphological features of the gels generated from the chaotropic salt **9:K**. Imaging, using SEM, of the xerogel formed from the **9:K***i*-PrOH gel identified an entangled web of fibrils with average diameter ≈ 200 nm (shown in Fig. 4a, see also ESI section S4†) and such morphology is typical for gelatinous materials. While the fibres identified from the gel formed in *n*-BuOH (Fig. 4b) appeared more linear and needle-like, the typical entangled morphology was maintained.

The sodium salt **9:Na** was a potent organogelator (Table 1) and using the solid material (4 mg added to 200 μL of a solvent) robust soft-materials, that withstood inversion tests, were obtained from EtOH, *i*-PrOH, *n*-BuOH, THF and 1,4-dioxane, whereas weaker gels were observed to form in CHCl_3_ and CH_2_Cl_2_.

The gelation experiments were also carried out by using a 5 μL addition of a 3 M aqueous solution of **9:Na** (50 μg in 50 μL of H_2_O, see ESI section S3† for full details) to the organic solvents (1 mL) followed by sonication of the resulting mixture. Formation of gels in *i*-PrOH, THF and 1,4-dioxane was again observed at low gelator concentration (0.5 wt%). The formation of gels in both THF and 1,4-dioxane occurred extremely rapidly, especially in the case of THF, which was observed to gelate immediately after addition of the **9:Na** solution without the use of either heat or sonication to trigger the gelation process, again demonstrating that this salt could function as a potent LMIOG.

**Fig. 5:**
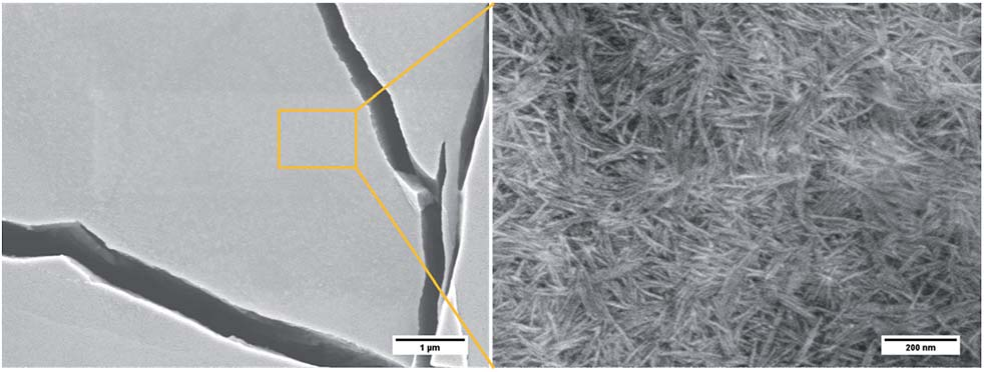
SEM images of xerogel obtained from **9:Na** EtOH gel (2 wt%).

The SEM images of the xerogels formed from the EtOH and *i*-PrOH gels were largely devoid of the long fibrils observed previously, instead a fibrous matlike residue was seen (Fig. 5). The lack of fibrils was believed to be due to the extremely rapid gelation process that occurred before extended fibril formation could take place.

**Fig. 6:**
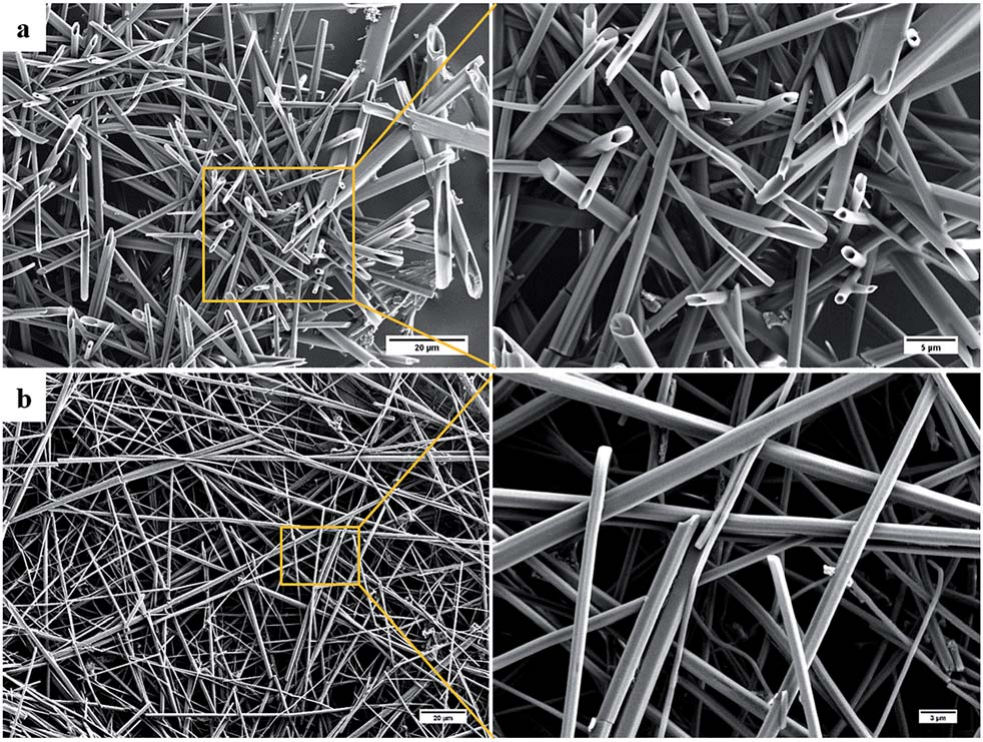
SEM images of **9:Na** xerogel obtained from (a) *i*-PrOH gel (1 wt%) showing hollow hexagons and (b) *n*-BuOH gel (1 wt%) showing rods.

When the SEM imaging experiments were performed using a larger sample of the *i*-PrOH gel (20–30 μL) hollow hexagonal columnar structures (1–3 μm across) were observed in the resulting xerogel (Fig. 6). Recently, the group of Hardie identified a similar architecture from the crystallisation of a large bowl shaped cyclotriveratrylene (*M*_W_ 723.74) with CuBr_2_,^58^ and previous work by Li and co-workers revealed that a structurally similar compound, diphenylalanine, was also capable of assembly into hollow hexagonal crystal.^59^ Hollow hexagonal crystals were also reported from the self-assembly of cyclic peptides in a liquid crystal by Dory *et al.*^60^ Very recently, Pasán *et al.* reported the use of Ostwald ripening to grow ∼300 μm sized hollow crystals, from a planar trinuclear Cu(ii)–cyamelurate complex.^61^ The crystals identified by Pasán possessed hollow hexagonal prismatic morphology, very similar to that identified here, demonstrating the growth of larger sized (micron) hollow tubular structures from a bulk solution.^61^ Nevertheless, such nanometre-scale morphology is remarkably rare and to the best of our knowledge, the work presented herein, is the first instance where such topology has been observed in a xerogel formed from LMIOGs. The hollow hexagons were reproducibly formed from *i*-PrOH at a range of gelator concentrations (0.5–2 wt%). These organogels were found to be stable over several months, and no change was observed in their morphological features when SEM images were subsequently recorded.

**Fig. 7:**
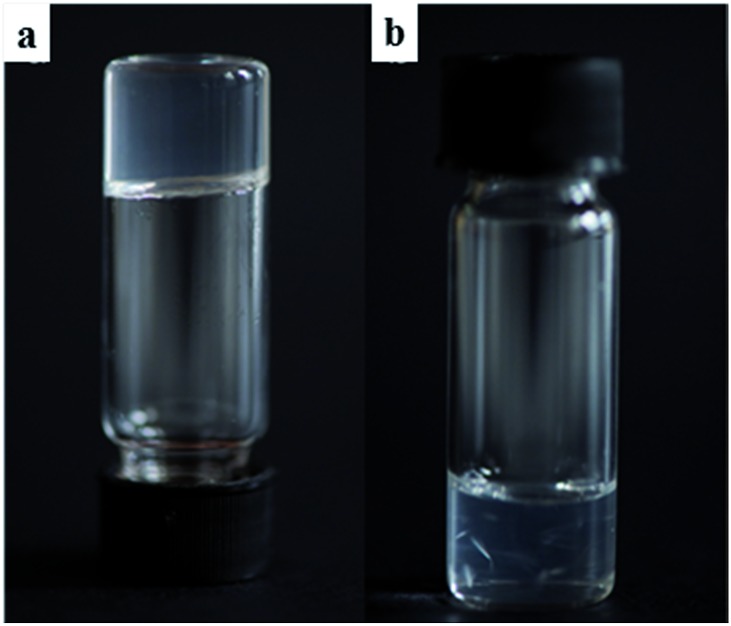
(a) A clear gel formed using **9:Na** in *i*-PrOH (1 wt%); (b) crystals of **9:Na** forming in a 1,4-dioxane gel (1 wt%) after 7 days.

These hollow tubes were not observed in any other solvent indicating a remarkable solvent effect on the nanoscale morphology. In *n*-BuOH (Fig. 6) similar intertwined rods (0.5–2 μm) formed; yet in comparison to the hollow hexagons systems, these appeared more solid, narrower, and somewhat flexible. Gels from **9:Na** in hydrophilic solvents, especially *i*-PrOH and *n*-BuOH, were stable to the inversion test for periods of several weeks before fine needle-like crystals became evident (Fig. 7) eventually leading to gel collapse. Gels in volatile hydrophobic solvents, especially CH_2_Cl_2_ and CHCl_3_, were not as stable, lasting only a few days (unless carefully sealed to prevent solvent evaporation) before crystals formed and the gel collapsed.

**Fig. 8:**
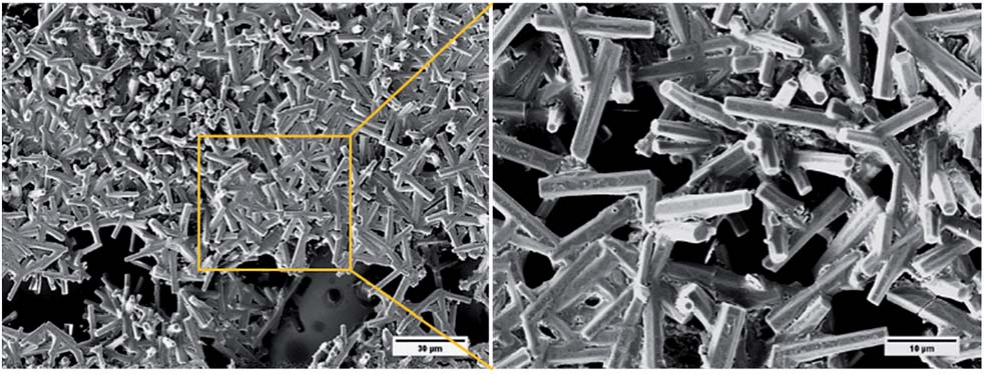
SEM images of **9:Li** xerogel from 1,4-dioxane (2 wt%).

In contrast to these results, for the lithium salt **9:Li**, no gels could be reliably obtained, although evidence of aggregation was observed in 1,4-dioxane solution at slightly higher concentration (2 wt%) and only if this hygroscopic salt was rigorously dried prior to the gelation tests, ruling out the use of this system as a LMIOG. When this aggregate was examined using SEM, unique hexagonal microcrystals (*ca.* 15 × 3 μm) were revealed (Fig. 8).

The structural variation in xerogel morphology clearly indicates the role of both the counterions and the solvent. Unfortunately, the tetraalkylammonium salts of **9** proved to be very hygroscopic, and consequently, none were capable of initiating a biphasic system nor the formation of a soft-material in any solvent, instead these tetraalkylammonium salts were readily soluble in most of the organic solvents tested (see ESI section S3† for details of evaluation).

### Varying the gelator structure

**Fig. 9:**
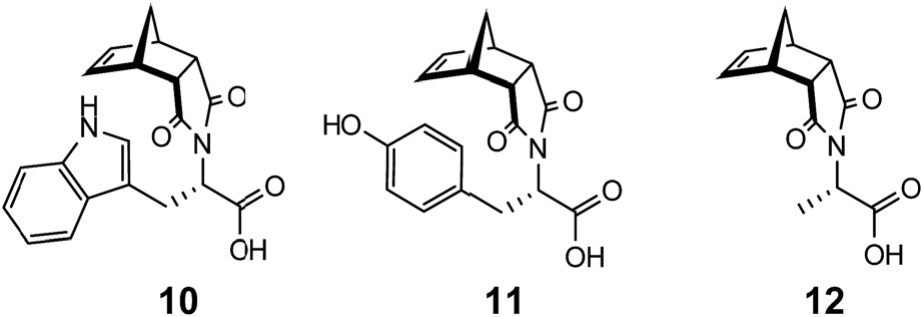
Tryptophan, tyrosine and alanine functionalised norbornenes **10–12**.

In addition to examining the role of the counterion in gel formation, additional structural modifications using amino acids were pursued. The tryptophan (**10**), tyrosine (**11**) and alanine (**12**) analogues of **9** (Fig. 9) were all readily synthesised (see ESI section S1† for details). While the tryptophan functionalised norbornene **10** formed a triphasic system when washed with a saturated Na_2_CO_3_ solution, the isolated sodium salt **10:Na** failed to elicit gel formation in any solvent trialled. Similarly, both **11:Na** and **12:Na** showed similar behaviour and were not able to function as LMIOG.

**Fig. 10:**
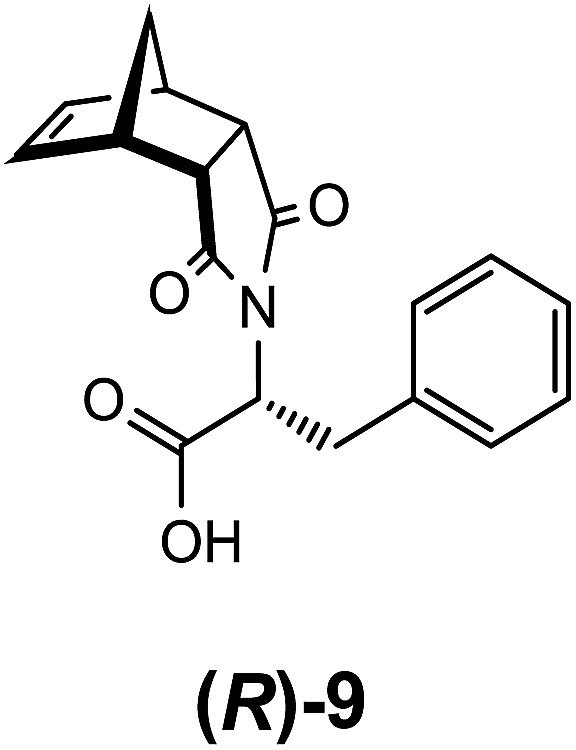
(*R*)-Phenylalanine functionalised norbornene **(*R*)-9**.

To investigate the effects of chirality on the self-assembly and gelation process, (*R*)-phenylalanine (Fig. 10) was also synthesised and used to generate **(*R*)-9:Na**. As expected, this enantiomer and its behaviour was identical in all aspects to the (*S*) stereoisomer including the formation of the biphasic system, confirming that this enantiomer could also function as a LMIOG. Of interest, it was noted that a racemic mixture of **(*R*)-9:Na** and **(*S*)-9:Na** failed to form gel material in any of the solvent systems employed above, indicating that the supramolecular assembly process leading to solvent entanglement possibly also relied on the presence of a single enantiomer.^8^,^62^–^66^

### Rheological evaluation

Having investigated the morphological features of the various xerogels, we set out to probe the mechanical properties of the gels formed by **9:K** and **9:Na** by carrying out dynamic rheological measurements, which included frequency and strain sweep and recovery tests. Prior to these investigations, differential scanning calorimetry (DSC) was performed on the gel samples and the results revealed that these gels had sharp gel–sol transition points 20–30 °C below the boiling point of the solvent (see ESI Table S5.1, section S5†).

**Fig. 11:**
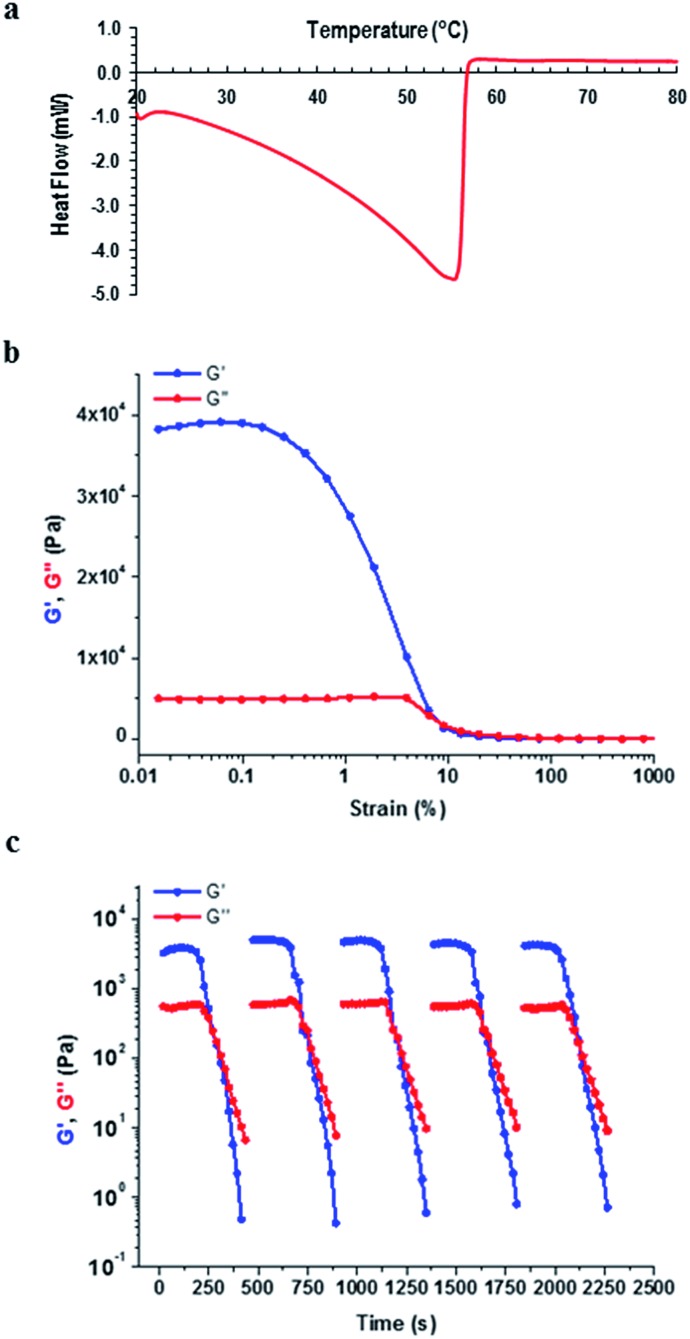
Characterisation of the 1 wt% gel formed by **9:Na** in *i*-PrOH (a) DSC (b) rheological strain sweep and (c) rheological recovery test.

Frequency sweep experiments showed elastic response in the linear viscoelastic regime, wherein, the storage modulus (*G*′) was consistently higher than the loss modulus (*G*′′) across all samples, as shown for the 1% **9:Na** gel formed in *i*-PrOH in Fig. 11 (see ESI† section S6† for all rheological results), confirming the solid like behaviour of the gels. Both the *i*-PrOH and *n*-BuOH gels of **9:K** (2 wt%) possessed comparable rheological behaviour; a result that correlated well with the SEM observations where both gels had similar morphological features. Both the elasticity (*G*′ = 1.35 × 10^4^ and 7.3 × 10^4^ Pa for 2 wt% *i*-PrOH and *n*-BuOH gels respectively) and the stiffness (*G*′/*G*′′ = 6.2 and 7.3 for 6.2 for *i*-PrOH and *n*-BuOH) were similar, though the *n*-BuOH gel was found to be slightly stronger, as summarised in Table 2. The yield stress for both gels were also similar (σ* ∼ 10%). As **9:Na** formed gels in a larger assortment of organic solvents, a broader range of rheological behaviour was observed (Table 2). Typically the **9:Na** gels were stronger than their **9:K** counterparts, however, gel strength did not show any particular relationship with the solvent used.

**Table 2:** Dynamic rheology results for **9:K** and **9:Na** gels^*a*^

Gelator (wt%)/solvent	*G*′ (Pa)	Stiffness (*G*′/*G*′′)	σ* (Pa)
**9:K** (2 wt%)/*i*-PrOH	1.4 × 10^4^	6.2	9.4%
**9:K** (2 wt%)/*n*-BuOH	7.3 × 10^4^	7.3	9.8%
**9:Na** (2 wt%)/EtOH	6.9 × 10^3^	7.7	5.6%
**9:Na** (1 wt%)/*i*-PrOH	3.8 × 10^4^	7.6	7.5%
**9:Na** (2 wt%)/*i*-PrOH	1.1 × 10^5^	10.1	15%
**9:Na** (2 wt%)/*n*-BuOH	2 × 10^4^	3.8	8.6%
**9:Na** (2 wt%)/1,4-dioxane	1.2 × 10^5^	4.8	4.4%
**9:Na** (2 wt%)/CHCl_3_	1.6 × 10^5^	4.6	4.5%
**9:Na** (2 wt%)/THF	1.7 × 10^5^	3.2	0.4%

^*a*^ *G*′, *G*′′ and σ* values were obtained from strain sweep experiments performed at fixed frequency of 1 Hz.

**Fig. 12:**
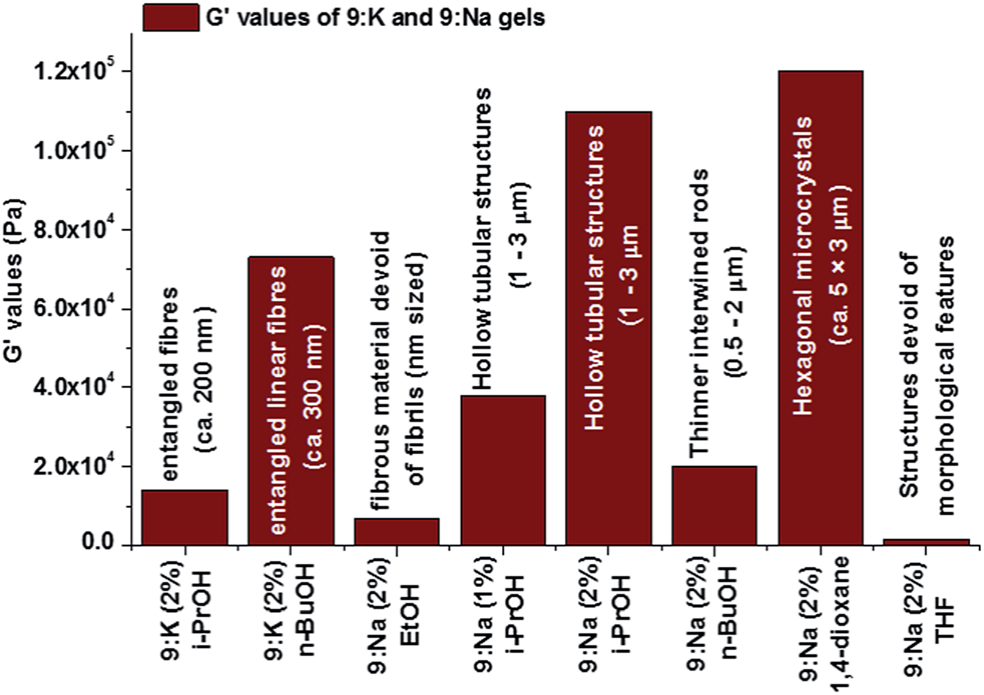
The variation of *G*′ values of **9:K** and **9:Na** gels and comparison to the morphology of the corresponding gels.

As mentioned above, the stiffness (*G*′/*G*′′) and yield strain (σ*) values correlated well to the morphological features of the xerogels that were observed using SEM (Fig. 12), and such a relationship suggests that the fibrils may be organised in similar arrangements in the corresponding gels. The lower *G*′ value for EtOH gel is likely to be due to the formation of smaller fibrils that lack the extended entanglement. In contrast, for the stronger **9:Na***i*-PrOH gel, the larger micrometre sized (1–3 μm diameter) hollow columnar structures in its gel matrix (*vide supra*) are likely contributors to the greater overall gel strength.

Both **9:K** and **9:Na** gels showed remarkable recovery properties; for the **9:Na** gel in *i*-PrOH (1 wt%) (Fig. 11c) applying a strain amplitude of 100% resulted in liquid like behaviour (*G*′′ > *G*′). However, the original gel state (*G*′ > *G*′′) instantly recovered when the strain was reduced to 0.1%. Recovery was observed over multiple cycles of measurements and all **9:K** and **9:Na** gels showed similar recovery behaviour within the 30–40 second interval between alternating strain regimes (see ESI section S6† for full details). These results demonstrate the thixotropic properties of the gels generated from these LMIOGs and suggest their potential as self-healing materials.

### Supramolecular assembly and crystal structure

**Fig. 13:**
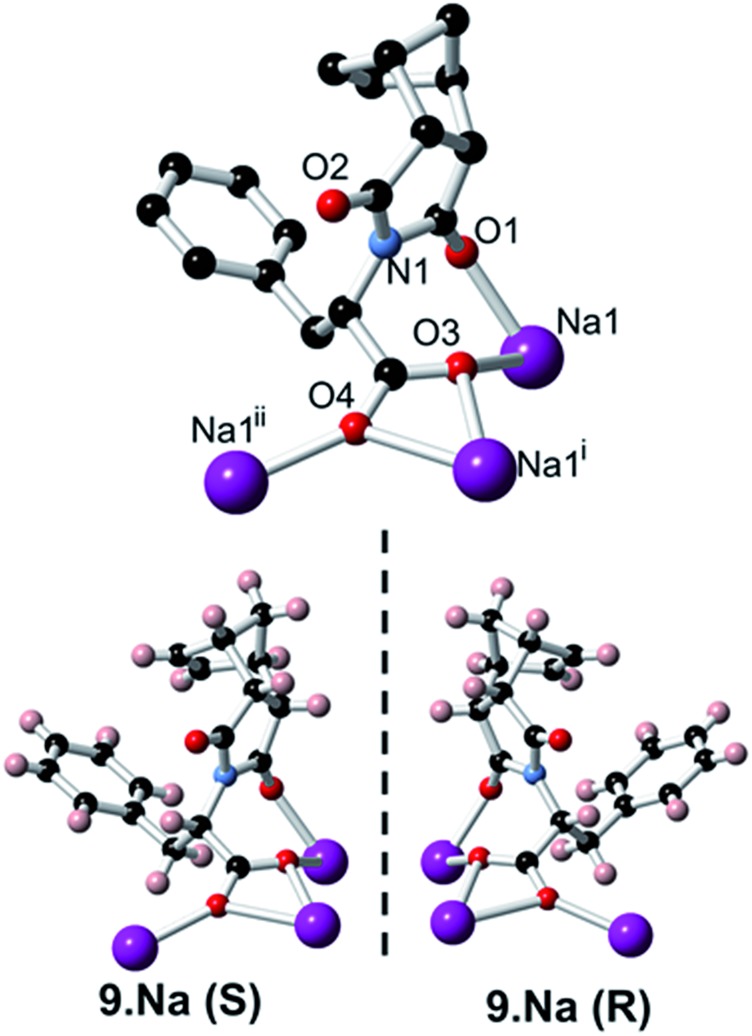
Crystal structure of both **(*S*)-9:Na** and **(*R*)-9:Na**.

In addition to carrying out the investigation into the ability of these LMIOGs to form soft-materials, we were also able to form crystalline material from these gels. Long needle-like crystals of **(*S*)-9:Na** that were suitable for X-ray analysis were obtained upon crystallisation from its *i*-PrOH gel (Fig. 7) and revealed that individual units of the norbornene adopt an amphiphilic structure in the solid state, with the norbornene framework and the phenylalanine aromatic positioned in close proximity (potentially through C(sp^2^)–H···π interactions)^67^–^69^ and the carboxylate residue oriented away from the hydrophobic portions (Fig. 13). This conformational arrangement was also evident in solution as the ^1^H NMR spectrum of **9** (see ESI Fig. S1.1†) revealed one of the norbornene C(sp^2^)–H resonances significantly upfield (*δ* = 5.33 ppm) from the other (*δ* = 5.62 ppm). Such a shift is indicative of anisotropic shielding due to the proximity of the aromatic ring current from the closely positioned phenylalanine residue.^70^,^71^

Using identical conditions, the structure of the **(*R*)-9:Na** enantiomer was also characterised and, as predicted, revealed the structural mirror image (Fig. 13). The racemate showed no evidence of gelation or crystal formation.

**Fig. 14:**
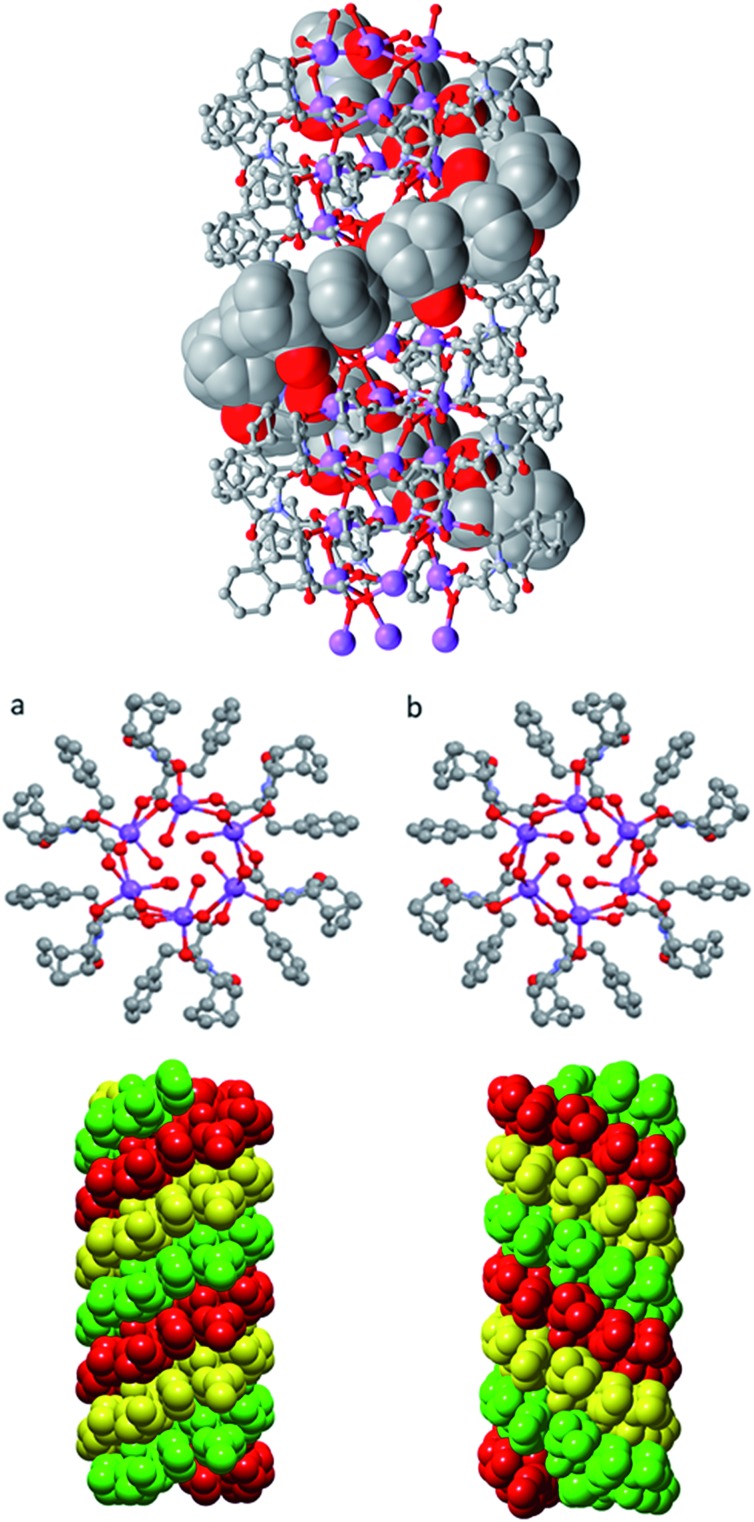
Helical columns formed by the self-assembly of (a) **(*S*)-9:Na** and (b) **(*R*)-9:Na** in *i*-PrOH observed in the crystal structure.

Further investigation into the long range interactions in the crystal structures revealed that the individual monomers assembled into hollow, helical columns (shown in Fig. 14a) with the helical assembly being mediated by the Na^+^ ions that adopt an irregular six-coordinate geometry. Four of these coordination sites are occupied by carboxylate groups with the final two sites being occupied by a water ligand and the imide oxygen *syn* to the chiral centre. It is this imide coordination that controls the direction of the helix, resulting in a right-handed conformation for **(*S*)-9:Na** and left-handed for **(*R*)-9:Na** (Fig. 14b).^72^ The helix has a pitch of 19.8 Å and a diameter of 19.6 Å, with 6 monomer residues per complete turn. Each additional monomer represents a 60° turn in the helix and a 3.2 Å translation along the helical axis.

**Fig. 15:**
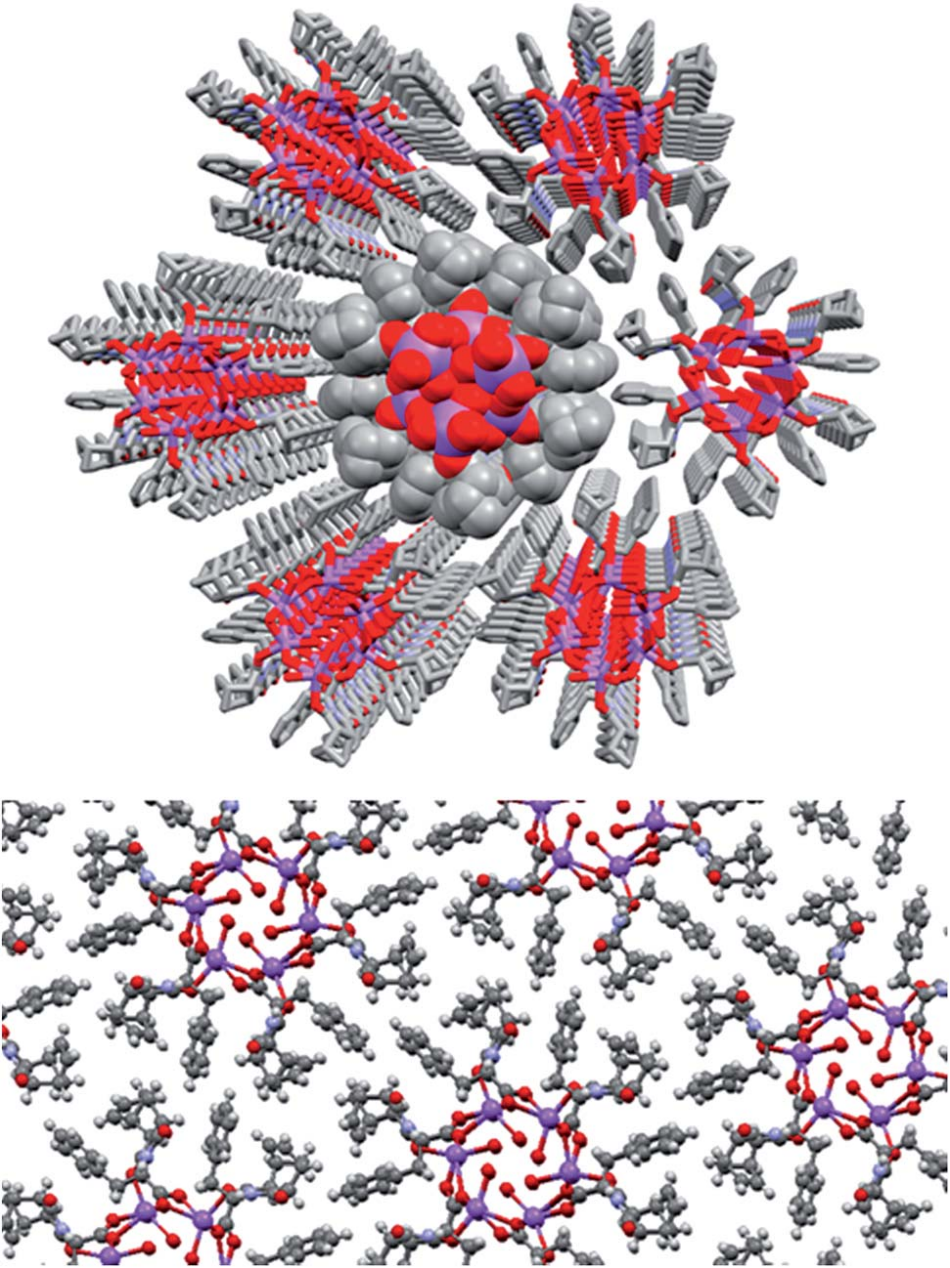
The full crystal network of **(*S*)-9:Na** showing two views of the repeating columnar helical assemblies and their interactions.

Adjacent stacks in the structure of **(*S*)-9:Na** associate in a hexagonal rod packing fashion with hydrophobic interactions between the outwards facing organic periphery (Fig. 15). No significant voids were observed in the interstitial spaces, indicating efficient packing of the homochiral helices. This observation offers some justification as to the need for enantiomeric purity as given the significant undulation in the external surface of the columns and the numerous intermolecular contacts (clearly visible using Hirshfeld surface mappings, see ESI, Fig. S7.3†), co-crystallisation of helices with alternate handedness would be expected to lead to a significant decrease in packing efficiency.

As a model for assembly, the largely hydrophobic structure of **9** protects the hydrophilic carboxylate by assembling such that the carboxylate is shielded from the organic solvent. As the amphiphile is chiral, a helical assembly occurs in which all carboxylates are oriented to the interior of the helix ensuring that they are not solvent exposed.

**Fig. 16:**
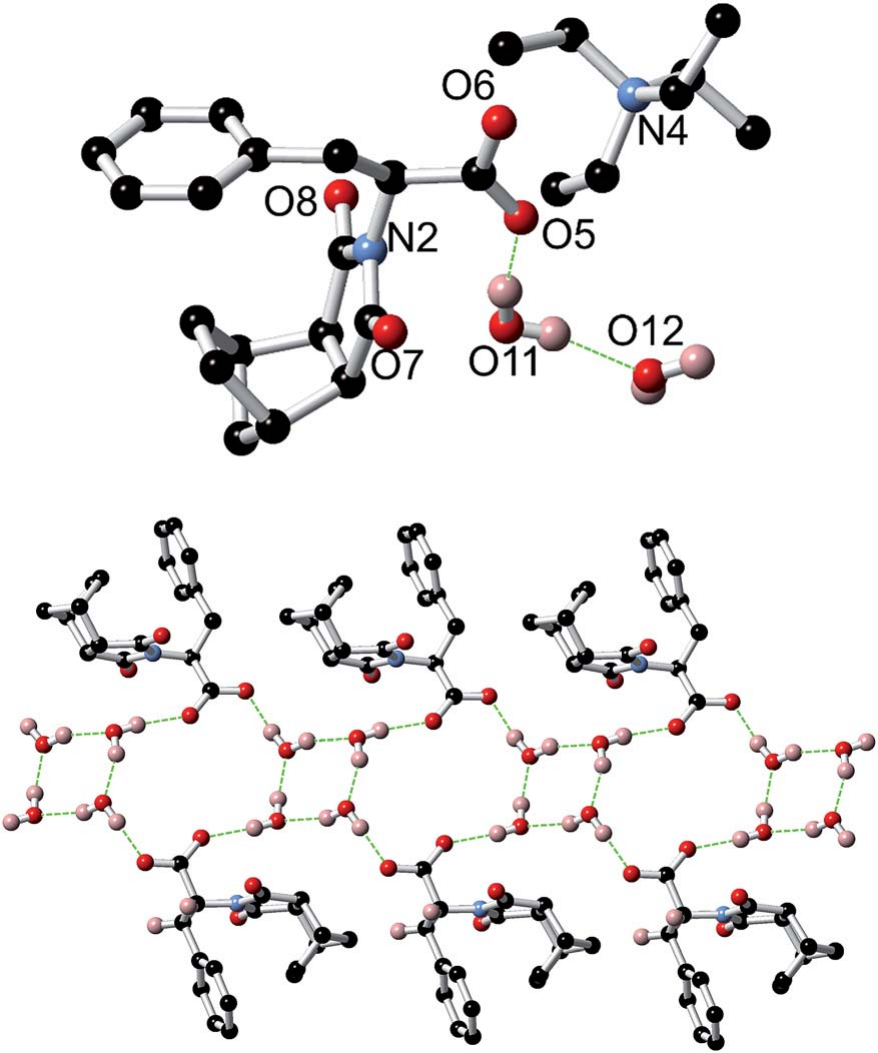
Top: Crystal structure of **9:TEA** showing the loose association of the TEA counterion. Non-bonding hydrogens have been omitted for clarity. Bottom: Crystal structure of **9:TEA** highlighting both the ribbon structure and the role of water in the assembly. The TEA counterions and non-bonding hydrogens have been omitted for clarity.

A crystal structure of the non-gelating salt **(*S*)-9:TEA** was also obtained and revealed the formation of thin ribbons (Fig. 16). In this example the counterions did not take part in the formation of the ribbons, instead only being loosely associated with the carboxylate moieties, demonstrating the importance of the small sodium cation in the helical assembly process for **9:Na**. Of additional interest, the X-ray structure of **(*S*)-9:TEA** revealed alternating cyclic tetramers of water and loops involving both water molecules and the carboxylate receptor as demonstrated in Fig. 16.^73^–^77^

## Conclusions

The results presented here show that compound **9:Na** is a highly capable small molecule . This ionic compound can readily form biphasic and triphasic systems and, in the solid state, assembles to form chiral helical columns. In addition, **9:Na** acts as an efficient low molecular weight ionic organogelator (LMIOG) in a variety of organic solvents at concentrations as low as 0.5 wt%. The rheological measurements of the gels demonstrate rapid recovery and that these organogels have potential as self-healing materials. Such interesting behaviour reinforces the importance of supramolecular assembly as a means to control macroscopic behaviour and also the requirement for careful characterisation to aid in the understanding of fundamental molecular-level interactions. We are in the process of developing other LMIOGs and investigating their properties and applications.

## Conflicts of interest

The authors declare no conflicts of interest.

## Supplementary Material

Supplementary information

Crystal structure data
