# Supplementary material for: Norbornene chaotropic salts as low molecular mass ionic organogelators (LMIOGs)
Source: Chem Sci. 2018 May 16;9(23):5233–41. doi: 10.1039/c8sc01798k (PMC6001280; doi:10.1039/c8sc01798k)
Supplement: Supplementary file 1 [file SC-009-C8SC01798K-s001.pdf]

Electronic Supplementary Material (ESI) for Chemical Science.
This journal is © The Royal Society of Chemistry 2018

# Norbornene chaotropic salts as low molecular mass ionic organogelators (LMIOGs)

Jordan R. Engstrom,[a] Aramballi J. Savyasachi,[b] Marzieh Parhizkar,[c] Alessandra Sutti,[c] Chris S. Hawes,[d] Jonathon M. White,[e] Thorfinnur Gunnlaugsson[b]* and Frederick M. Pfeffer[a]*

a School of Life and Environmental Sciences, Deakin University, Waurn Ponds, Vic, 3216, (Australia). email: fred.pfeffer@deakin.edu.au

b Trinity Biomedical Sciences Institute, Trinity College Dublin, University of Dublin, Dublin 2 (Ireland). email: gunnlaut@tcd.ie

c Institute for Frontier Materials, Deakin University, Waurn Ponds, Vic, 3216, (Australia).

d School of Chemical and Physical Sciences, Keele University, Staffordshire, ST5 5BG (UK).

e Bio21 Institute, School of Chemistry, University of Melbourne, Parkville, 3010 (Australia).

CONTENTS

**S1. Synthesis** ..... 2

**S2. Diffusion** ..... 8

**S3. Gelation** ..... 9

**3.1. Solid gelator** ..... 9

**S3.2. Gelator solution** ..... 16

**S4. SEM images** ..... 17

**S5. DSC calorimetry** ..... 22

**S6. Rheology** ..... 29

**S7. Crystallography** ..... 34

**8. References** ..... 38

## S1. Synthesis

### Reagents

All listed reagents were obtained from specialist suppliers and use without further purification. Pet. Spirits refers to the fraction of Petroleum Spirits boiling between 40 - 60 °C.

### Instrumentation

All $^1$H NMR and $^{13}$C NMR spectra were recorded on either a JEOL JNM-EX 270 MHz FT-NMR, a JEOL JNM-Eclipse+ 400 MHz FT-NMR or a as indicated. Samples were dissolved in deuterated chloroform ($CDCl_3$) and the relevant solvent peak ($CDCl_3$: δH 7.26 ppm) used as an internal reference. The template for reporting proton spectra is as follows: chemical shift δ (ppm), (multiplicity (s = singlet, br s = broad singlet, d = doublet, t = triplet, q = quartet, m = multiplet), coupling constant *J* (Hz), integral, assignment). All IR spectra were recorded using a Bruker ALPHA FTIR spectrometer in the range of 4000-500 $cm^{-1}$. The TGA experiments were performed using a TA Instruments TGA Q50 V20.13 Build 39. For the crystals of **9**:**Na** the heating was performed using a platinum pan, under nitrogen gas, at a heating rate of 10 degrees per minute up to 400 degrees.

### General procedure for synthesis of amino acid functionalised norbornenes

Norbornene anhydride (1 equiv.), amino acid (1.1 equiv.) and $NEt_3$ (0.4 equiv.) were combined in a round bottom flask with PhMe (10 mL per 1 g of anhydride). The reaction setup was fitted with a Dean-Stark apparatus and heated at reflux for 16 h. After this time, the reaction mixture was allowed to cool before the solvent was removed under reduced pressure. The residue was then taken up in EtOAc and transferred to a separatory funnel before being washed with 0.2M HCl (2 × 15 mL) and sat. NaCl (2 × 15 mL) before being dried ($MgSO_4$), filtered and the solvent removed under reduced pressure.

### Nomenclature and NMR assignment

All norbornane and fused [n]polynorbornane-based compounds are named according to the Von-Baeyer system of nomenclature as (multiplier)cyclo[X,Y,Z]hydrocarbon where X ≥ Y > Z > 0.[1] The stereodescriptors syn/anti are used to refer to substituents on the Z bridge that are located on the same/opposite side as the X bridge. The stereodescriptors α/β are used to refer to the configuration of substituents on the X and Y bridges. The norbornane system is numbered to allow for the longest possible chain before numbering bridges.

The amino acid side chain is assigned and labelled following Pople notation for AMX pairs.[2]

### (1α,2α,6α,7α)-4-[(*S*)-Carboxymethyl-1'-benzyl]-4-azatricyclo[5.2.1.0$^{2,6}$]deca-8-ene-3,5-dione [(*S*)-9][3]

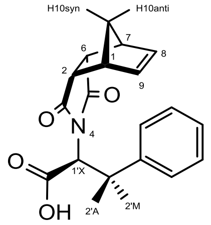


Following the general procedure, norbornene anhydride (3.0 g, 18.3 mmol), L-phenylalanine (3.3 g, 20.1 mmol), $NEt_3$ (1.5 mL, 10.8 mmol) and PhMe (30 mL) was refluxed for 16 hours. The pure product was obtained as an opaque amorphous solid (4.4 g, 77%).

**$^1$H NMR** (500 MHz, $CDCl_3$): δ 7.23–7.02 (m, 5H, Ar), 5.62 (d, *J* = 2.5 Hz, 1H, H9), 5.33 (s, 1H, H8), 4.96 (dd, *J* = 11.9, 5.0 Hz, 1H, H1'X), 3.40 (dd, *J* = 14.5, 5.0 Hz, 1H, H2'A), 3.27 (dd, *J* = 14.5, 11.9 Hz, 1H, H2'M),

3.18–3.09 (m, 3H, H1,2,7), 3.04 (dd, *J* = 7.1, 4.6 Hz, 1H, H6), 1.51 (d, *J* = 8.7 Hz, 1H, H10anti), 1.35 (d, *J* = 8.7 Hz, 1H, H10syn);*

**$^{13}$C NMR** (126 MHz, $CDCl_3$): δ 177.3, 177.1, 173.1, 136.3, 134.3, 134.1, 129.1, 128.4, 126.9, 52.7, 52.1, 45.9, 45.7, 44.9, 44.6, 33.6;

* Due to overlapping peaks, not all J couplings could be determined. All correlations have been confirmed through 2D-NMR spectroscopy (COSY, HSQC and HMBC experiments) and all spectra are in accordance with those previously reported.[3]

**(1α,2α,6α,7α)-4-[(*S*)-Carboxymethyl-1'-((*1H*-indol-3''-yl)methyl)]-4-azatricyclo[5.2.1.0$^{2,6}$]deca-8-ene-3,5-dione [10]**[4]

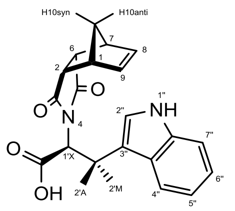


Following the general procedure, norbornene anhydride (3.0 g, 18.3 mmol), L-tryptophan (4.1 g, 20.1 mmol), $NEt_3$ (1.5 mL, 10.8 mmol) and PhMe (30 mL) was refluxed at 110 °C for 16 hours. The crude material was recrystallised from EtOAc and Pet. Spirits to give a dark maroon precipitate that was collected by means of vacuum filtration (4.8 g, 75%).

**$^{1}$H NMR** (500 MHz, $CDCl_3$) δ 8.39 (s, 1H, NH), 7.45 (d, *J* = 8.0 Hz, 1H, H7''), 7.24 (d, *J* = 8.0 Hz, 1H, H4''), 7.08 (dd, *J* = 11.1, 3.8 Hz, 1H, H5''), 7.02 (dd, *J* = 11.1, 3.8 Hz, 1H, H6''), 6.95–6.87 (m, 2H, H2'' and OH) 5.36 (dd, *J* = 5.4, 2.7 Hz, 1H, H9), 5.27 (dd, *J* = 5.4, 2.7 Hz, 1H, H8), 4.99 (dd, *J* = 10.3, 6.5 Hz, 1H, H1'X), 3.65 (app. q, *J* = 6.5 Hz, 1H, H2'A), 3.52–3.41 (m, 1H, H2'M), 3.12–3.08 (m, 2H H1 and H7), 3.02 (dd, *J* = 7.4, 4.5 Hz, 1H, H2), 2.88 (dd, *J* = 7.4, 4.5 Hz, 1H, H6), 1.44 (d, *J* = 9.5 Hz, 1H, H10anti), 1.24 (d, *J* = 9.5 Hz, 1H, H10syn).*

**$^{13}$C NMR** (126 MHz, $CDCl_3$) δ 177.6, 177.6, 136.1, 134.0, 133.8, 127.5, 123.1, 122.0, 119.4, 118.6, 111.3, 110.5, 58.4, 52.5, 52.1, 46.0, 45.7, 44.9, 44.7, 18.0.

* Due to overlapping peaks, not all J couplings could be determined. All correlations have been confirmed through 2D-NMR spectroscopy (COSY, HSQC and HMBC experiments) and all spectra are in accordance with those previously reported.[4]

**(1α, 2α,6α,7α)-4-[(S)-Carboxymethyl-1'-(4''-hydroxybenzyl)]-4-azatricyclo[5.2.1.0$^{2,6}$]deca-8-ene-3,5-dione [11]**

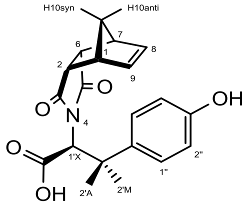


Following the general procedure, norbornene anhydride (2.0 g, 12.2 mmol), L-tyrosine (2.43 g, 13.4 mmol), $NEt_3$ (2.1 mL, 17.1 mmol) and PhMe (20 mL) was refluxed at 110 °C for 12 hours. The crude material was recrystallised from $CHCl_3$ to give yellow crystals that were collected by means of vacuum filtration (2.71 g, 67%).

**mp**: 101.5 – 107.9 °C

**$^{1}$H NMR** (500 MHz, DMSO-$d_6$): δ 11.59 (s, 1H, COOH), 7.32 – 7.10 (m, 5H, ArH × 4 & ArOH), 5.70 (dd, *J* = 5.4, 2.6 Hz, 1H, H9), 5.38 (d, *J* = 4.0 Hz, 1H. H8), 5.06 (dd, *J* = 11.7, 5.1 Hz, 1H, H1'X), 3.49 (dd, *J* = 14.7, 4.9 Hz, 1H, H2'A), 3.34 (dd, *J* = 14.5, 11.9 Hz, 1H, H2'M), 3.21 (dd, *J* = 11.8, 4.7 Hz, 3H, H1,2 and 7), 3.12 (dd, *J* = 6.6, 4.9 Hz, 1H, H6), 1.58 (d, *J* = 8.6 Hz, 1H, H10anti), 1.43 (d, *J* = 8.6 Hz, 1H, H10syn).*

**$^{13}$C NMR** (67.5 MHz; DMSO-$d_6$): δ 177.1, 176.9, 170.3, 156.3, 134.7, 134.1, 130.3, 127.4, 115.3, 79.6, 53.1, 52.0, 45.5, 45.4, 32.87, 31.2;

**HRMS** *m/z* (ESI): 328.1179 ($C_{18}H_{18}NO_5$ $[M + H]^+$ requires 328.1185

* Due to overlapping peaks, not all J couplings could be determined. All correlations have been confirmed through 2D-NMR spectroscopy (COSY, HSQC and HMBC experiments).

**(1α, 2α,6α,7α)-4-[(*S*)-Carboxymethyl-1'-methyl]-4-azatricyclo[5.2.1.0$^{2,6}$]deca-8-ene-3,5-dione [12][5]**

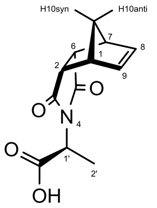


Following the general procedure, norbornene anhydride (1.0 g, 6.1 mmol), L-alanine (270 mg, 6.1 mmol), $NEt_3$ (0.5 mL, 3.6 mmo) and PhMe (20 mL) was refluxed at 110 °C for 12 hours. The crude material was recrystallised from EtOAc and Pet. Spirits to give pure white crystals that were collected by means of vacuum filtration (990 mg, 79%).

**mp**: 143.6 – 149.5 °C

**$^1$H NMR** (500 MHz, $CDCl_3$): δ 9.12 (s, 1H, COOH), 6.14–6.10 (m, 2H, H8,9), 4.69 (q, *J* = 7.4 Hz, 1H, Hα), 3.46–3.38 (m, 2H, H1,7), 3.33 (qd, *J* = 7.4, 4.3 Hz, 2H, H2,6), 1.75 (dt, *J* = 8.8, 1.5 Hz, 1H, H10anti), 1.56 (d, *J* = 8.8 Hz, 1H, H10syn) 1.44 (d, *J* = 7.2 Hz, 3H, H2');*

**$^{13}$C NMR** (67.5 MHz; $CDCl_3$): δ 176.8, 176.7, 174.5, 134.6, 134.3, 52.2, 47.3, 45.9, 45.7, 45.2, 45.0, 14.3;

* Due to overlapping peaks, not all J couplings could be determined. All correlations have been confirmed through 2D-NMR spectroscopy (COSY, HSQC and HMBC experiments) and all spectra are in accordance with those previously reported.[5]

**(1α,2α,6α,7α)-4-[(*R*)-Carboxymethyl-1'-benzyl]-4-azatricyclo[5.2.1.0$^{2,6}$]deca-8-ene-3,5-dione [(*R*)-9][6]**

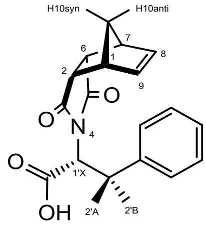


Following the general procedure, norbornene anhydride (2.0 g, 12.2 mmol), D-phenylalanine (2.2 g, 13.1 mmol), $NEt_3$ (1.0 mL, 7.2 mmol) and PhMe (20 mL) was refluxed at 110 °C for 12 hours. The pure product was obtained as an opaque amorphous solid (2.33 g, 62%).

**$^1$H NMR** (500 MHz, $CDCl_3$): δ 7.23–7.02 (m, 5H, Ar), 5.62 (d, *J* = 2.5 Hz, 1H, H9), 5.33 (s, 1H, H8), 4.96 (dd, *J* = 11.7, 5.1 Hz, 1H, H1'X), 3.40 (dd, *J* = 14.7, 5.0 Hz, 1H, H2'A), 3.27 (dd, *J* = 14.3, 12.1 Hz, 1H, H2'M), 3.13 (dd, *J* = 13.7, 6.0 Hz, 3H, H1,2,7), 3.04 (dd, *J* = 7.1, 4.6 Hz, 1H, H6), 1.51 (d, *J* = 8.7 Hz, 1H, H10anti), 1.35 (d, *J* = 8.6 Hz, 1H, H10syn);*

**$^{13}$C NMR** (126 MHz, $CDCl_3$): δ 177.3, 177.1, 173.1, 136.3, 134.3, 134.1, 129.1, 128.4, 126.9, 52.7, 52.1, 45.9, 45.7, 44.9, 44.6, 33.6;

* Due to overlapping peaks, not all J couplings could be determined. All correlations have been confirmed through 2D-NMR spectroscopy (COSY, HSQC and HMBC experiments) and all spectra are in accordance with those previously reported.[6]

### General Procedure for salt formation

All sodium and potassium salts were formed by dissolving a suitable quantity of the acid in EtOAc and transferring to a separatory funnel. The organic phase was then extracted with excess sat. $Na_2CO_3$ or $K_2CO_3$. From the resultant triphasic mixture the middle layer was separated and concentrated to dryness. The resultant white solid was the desired salt (quantitative).

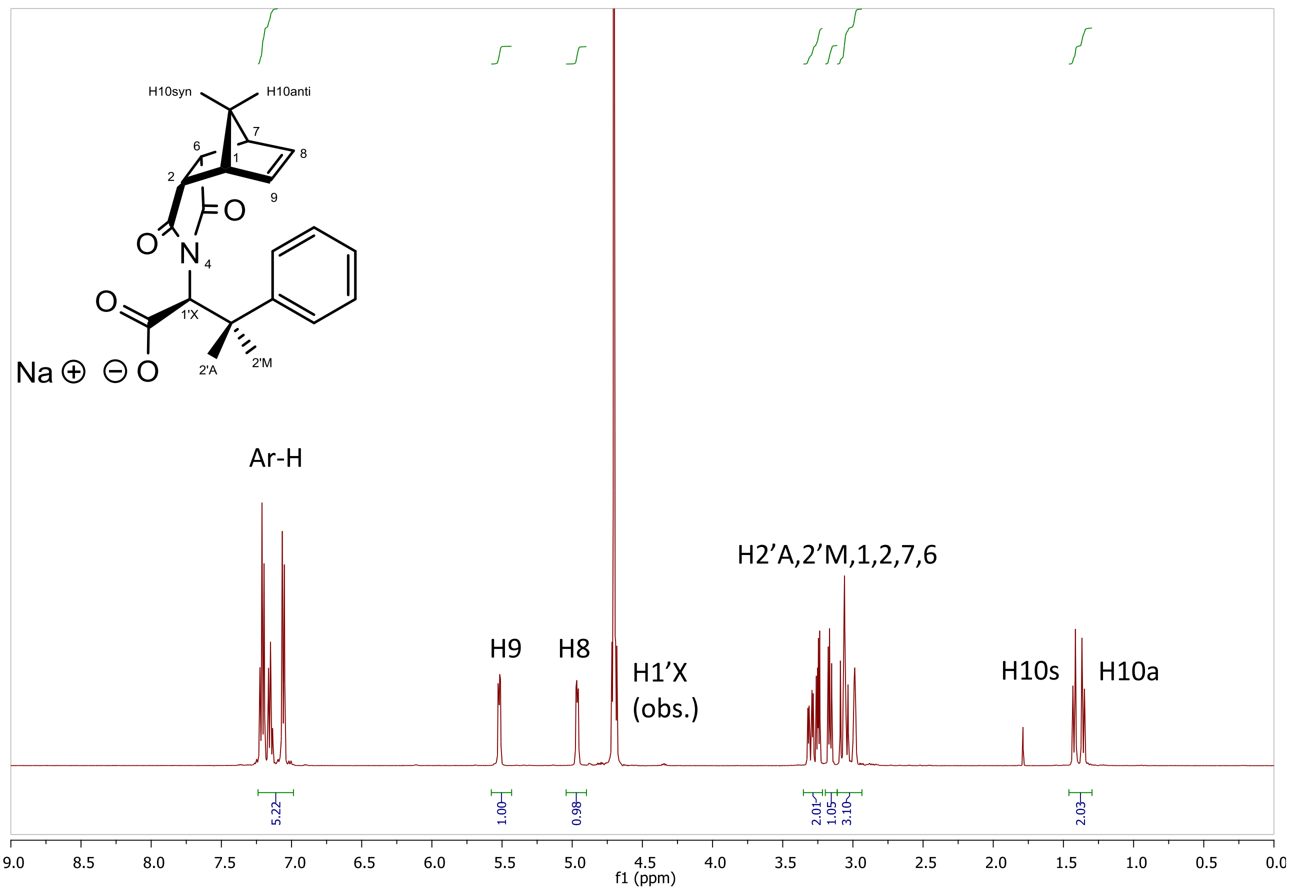


**Figure S1.1**: $^1$H NMR of (S)-9:Na in $D_2O$

All other salts were formed by dissolving **(*S*)-9** (1 equiv.) in *i*-PrOH and adding an aqueous solution of the cation hydroxide (1 equiv.). The combined solution was stirred overnight before being concentrated to dryness to afford the desired salt.

All salts were observed to be pure through $^1$H NMR spectroscopy

The melting point of all salts were observed to be over 300 °C (the limits of our instrumentation) except for **10:Na** which melted from 75.6 – 82.5 °C.

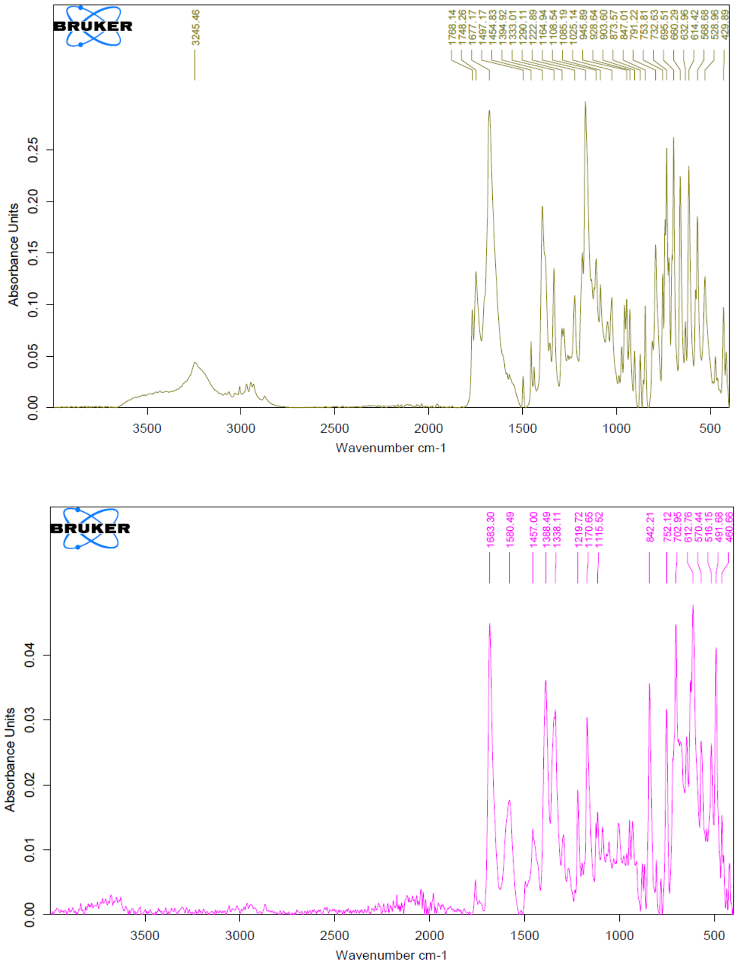


**Figure S1.2**: IR spectra of **9** (top) and **9:Na** (bottom).

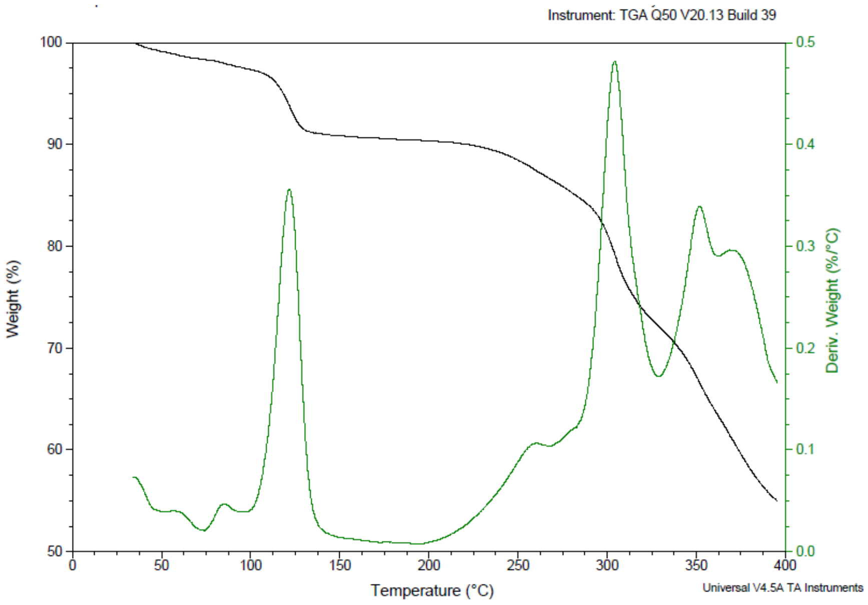


**Figure S1.3**: TGA analysis of **9**:**Na**. Black line indicates mass loss and the green line shows the 1st derivative of the mass loss curve.

**Aqueous biphasic system**

To identify compounds capable of forming aqueous biphasic systems an aqueous (5 M) solution of the compound was added to a test tube then the equivalent volume of sat. $Na_2CO_3$ added. After stoppering and shaking if the aqueous biphasic system was not immediately apparent the solution was heated to near boiling and upon cooling again inspected for a biphasic system.

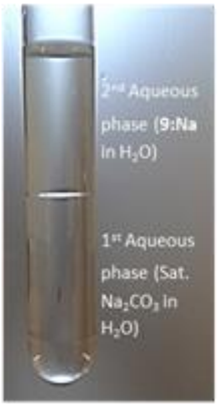


**Figure S1.4**: Aqueous biphasic system formed by combining equal volumes of aqueous **9:Na** (5M) and sat. aqueous $Na_2CO_3$.

## S2. Diffusion

$^1$H relaxation measurements were carried out at 11.7 T on a Bruker Avance III 500 MHz wide-bore spectrometer and a 5 mm static probe. Samples of **(S)-9:Na** were made up to the desired concentration in 500 μL of $D_2O$.

The pulsed field gradient stimulated echo (PFG-STE) pulse sequence was utilised with the magnitude of the applied gradient varied in 16 increments and the maximum gradient strength, gradient pulse length and diffusion time (D) optimised in each case. The 1H 90° pulse length was 9.5 ms and recycle delays were set to > 5 × $T^1$ in all cases. The data were fitted to the Stejskal–Tanner equation (Equation 1 below) using a non-linear least-squares method within the Bruker TopSpin software (v3):

$$I = I_0 exp\left[-D\gamma^2 g^2 \delta^2 \left(\Delta - \frac{\delta}{3}\right)\right] \qquad \textit{Equation 1}$$

In this expression, I is the observed signal intensity, $I_0$ is the maximum signal intensity, γ is the gyromagnetic ratio of the nucleus being observed, g is the gradient strength and δ is the gradient pulse duration.[7]

Diffusion constants were determined for multiple integral regions for each sample and averaged to provide the final diffusion constant plotted in Figure 2 in the manuscript.

**Table S2.1**. Diffusion rate of **9:Na** at different concentrations (× $10^{-10}$ $m^2/s$)

| | 0.5 mM | 1 mM | 2.5 mM | 5 mM | 10 mM | 15 mM | 20 mM |
|---|---|---|---|---|---|---|---|
| 7.3 - 6.9 ppm | 2.991 | 2.983 | 2.977 | 3.673 | 3.780 | 3.743 | 3.749 |
| 5.6 – 5.4 ppm | Obsc.[a] | Obsc.[a] | 2.944 | 3.687 | 3.68 | 3.695 | 3.732 |
| 5.1 – 4.9 ppm | Obsc.[a] | Obsc.[a] | Obsc.[a] | 3.870 | 3.743 | 3.604 | 3.819 |
| 3.4 – 2.9 ppm | 2.962 | 2.927 | 2.921 | 3.851 | 3.790 | 3.774 | 3.749 |
| 1.5 – 1.2 ppm | 3.00 | 2.965 | 2.952 | 3.829 | 3.785 | 3.782 | 3.727 |
| **Average** | **2.984 ± 0.117** | **2.958 ± 0.0165** | **2.949 ± 0.0116** | **3.782 ± 0.0422** | **3.756 ± 0.206** | **3.755 ± 0.0327** | **3.755 ± 0.0166** |

a) A reliable diffusion constant for these regions could not be determined due to the dilute nature of the sample and interference from the residual water peak.

## S3. Gelation

### 3.1. Solid gelator

Gelation tests were carried out by adding the gelator to the organic solvent as a solid powder before heating to the boiling temperature of the solvent (table S3.1). The solutions were then allowed to cool to room temperature. All apparent gels were then subjected to the six hour inversion test. If the six hour inversion test was passed, the gel classification was maintained. A turbid gel was designated to all gels not opaque in nature.

The following terms were used to classify the remaining samples:

Weak Gel: initial gel formation noted but the six hour inversion test was failed.
Partial Gel: the gel failed to entrap all the solvent.
Dissolved: sample was fully dissolved, resulting in a clear solution.
Insoluble: the sample failed to fully dissolve resulting in a precipitate.
Suspension: the sample remained dispersed throughout the solvent upon standing.
Aggregate: large accumulations of fine solid material encapsulated in small gel-like formations.

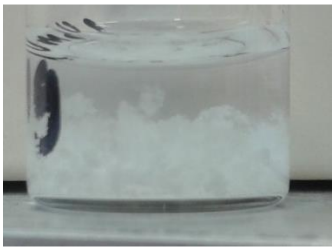

**Figure S3.1**. Photograph of aggregate formed when **9:Na** was mixed with acetonitrile.

Compounds **10-12** displayed no gelation behaviour in any of the tested solvents and so have been omitted from these results.

**Gelation of (*S*)-9:K**

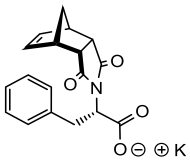


**(*S*)-9:K**

**Table S3.1.** Solvent gelation using **(*S*)-9:K** at 4 mg/200 μL (2 Wt%)

| Solvent | Experiment | Result |
|---|---|---|
| MeOH | Heated to 80°C | Dissolved |
| EtOH | Heated to 110°C | Dissolved |
| *i*-PrOH | Heated to 110°C | **Turbid Gel** |
| *n*-BuOH | Heated to 120°C | **Turbid Gel** |
| $CHCl_3$ | Heated to 85°C | Aggregate |
| $CH_2Cl_2$ | Heated to 85°C | Aggregate |
| THF | Heated to 85°C | Insoluble |
| $CH_3CN$ | Heated to 120°C | Aggregate |
| 1,4-dioxane | Heated to 120°C | Insoluble |
| Acetone | Heated to 75°C | Aggregate |
| EtOAc | Heated to 85°C | Aggregate |
| Toluene | Heated to 120°C | Suspension |
| Pet. Spirits | Heated to 90°C | Insoluble |
| Hexane | Heated to 90°C | Insoluble |
| DMF | Heated to 120°C | Dissolved |
| DMSO | Heated to 120°C | Dissolved |
| $H_2O$ | Heated to 110°C | Dissolved |

**Table S3.2.** Minimum gelation concentration of **(*S*)-9:K** in *i*-PrOH (1.0 mL).

| Wt of Comp | % Wt of comp | Experiment | Result |
|---|---|---|---|
| 20 mg | 2 wt % | Heated to 110 $^{o}$C | **Gel** |
| 10 mg | 1 wt % | Heated to 120 $^{o}$C | Dissolved |
| 7.5 mg | 0.75 wt% | Heated to 120 $^{o}$C | Dissolved |
| 5 mg | 0.5 wt% | Heated to 120 $^{o}$C | Dissolved |
| 2.5 mg | 0.25 wt% | Heated to 120 $^{o}$C | Dissolved |

**Table S3.3.** Minimum gelation concentration of **(*S*)-9:K** in *n*-BuOH (1.0 mL).

| Wt of Comp | % Wt of comp | Experiment | Result |
|---|---|---|---|
| 20 mg | 2 wt % | Heated to 120 $^{o}$C | **Gel** |
| 10 mg | 1 wt % | Heated to 120 $^{o}$C | Dissolved |
| 7.5 mg | 0.75 wt% | Heated to 120 $^{o}$C | Dissolved |
| 5 mg | 0.5 wt% | Heated to 120 $^{o}$C | Dissolved |
| 2.5 mg | 0.25 wt% | Heated to 120 $^{o}$C | Dissolved |

**Gelation of (*S*)-9:Na**

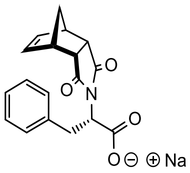


(*S*)-9:Na

**Table S3.4.** Solvent gelation using **(*S*)-9:Na** at 4 mg/200 µL (2 Wt%)

| **Solvent** | **Experiment** | **Result** |
|---|---|---|
| MeOH | Heated to 80°C | Dissolved |
| EtOH | Heated to 90°C | **Gel** |
| *i*-PrOH | Heated to 80°C | **Gel** |
| *n*-BuOH | Heated to 120°C | **Gel** |
| $CHCl_3$ | Heated to 70°C | **Gel** |
| $CH_2Cl_2$ | Heated to 70°C | Weak Gel |
| THF | Heated to 70°C | Suspension |
| $CH_3CN$ | Heated to 90°C | Aggregate |
| 1,4-dioxane | Heated to 100°C | Weak Gel |
| Acetone | Heated to 75°C | Partial Gel |
| EtOAc | Heated to 90°C | Partial Gel |
| Toluene | Heated to 110°C | Aggregate |
| Pet. Spirits | Heated to 75°C | Insoluble |
| Hexane | Heated to 75°C | Insoluble |
| DMF | Heated to 110°C | Dissolved |
| DMSO | Heated to 110°C | Dissolved |
| $H_2O$ | Heated to 100°C | Dissolved |

**Table S3.5.** Minimum gelation concentration of **(*S*)-9:Na** in EtOH (1.0 mL)

| **Wt of Comp** | **% Wt of comp** | **Experiment** | **Result** |
|---|---|---|---|
| 20 mg | 2 wt % | Heated to 105 °C | **Gel** |
| 10 mg | 1 wt % | Heated to 105 °C | Dissolved |
| 7.5 mg | 0.75 wt% | Heated to 105 °C | Dissolved |
| 5 mg | 0.5 wt% | Heated to 105 °C | Dissolved |
| 2.5 mg | 0.25 wt% | Heated to 105 °C | Dissolved |

**Table S3.6.** Minimum gelation concentration of **(*S*)-9:Na** in *i*-PrOH (1.0 mL).

| **Wt of Comp** | **% Wt of comp** | **Experiment** | **Result** |
|---|---|---|---|
| 20 mg | 2 wt % | Heated to 105 °C | **Gel** |
| 10 mg | 1 wt % | Heated to 105 °C | **Gel** |
| 7.5 mg | 0.75 wt% | Heated to 105 °C | **Gel** |
| 5 mg | 0.5 wt% | Heated to 105 °C | Weak gel |
| 2.5 mg | 0.25 wt% | Heated to 105 °C | Weak gel |

**Table S3.7.** Minimum gelation concentration in *n*-BuOH (1.0 mL).

| Wt of Comp | % Wt of comp | Experiment | Result |
|---|---|---|---|
| 20 mg | 2 wt % | Heated to 120 $^{o}$C | **Gel** |
| 15 mg | 1.5 wt % | Heated to 120 $^{o}$C | **Gel** |
| 10 mg | 1 wt% | Heated to 120 $^{o}$C | **Gel** |
| 7.5 mg | 0.75 wt% | Heated to 120 $^{o}$C | **Gel** |
| 5 mg | 0.5 wt% | Heated to 120 $^{o}$C | **Gel** |
| 2.5 | 0.25 wt% | Heated to 120 $^{o}$C | Dissolved |

**Gelation of (*S*)-9:Li**

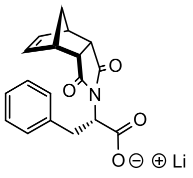


(*S*)-9:Li

**Table S3.8.** Solvent gelation using **(*S*)-9:Li** at 8 mg/400 µL (2 Wt%)

| Solvent | Experiment | Observation |
|---|---|---|
| MeOH | Heated to 80 °C | Insoluble |
| EtOH | Heated to 90 °C | Insoluble |
| *i*-PrOH | Heated to 90 °C | Insoluble |
| *n*-BuOH | Heated to 120 °C | Insoluble |
| $CHCl_3$ | Heated to 70 °C | Insoluble |
| $CH_2Cl_2$ | Heated to 70 °C | Insoluble |
| THF | Heated to 80 °C | Suspension |
| $CH_3CN$ | Heated to 110 °C | Insoluble |
| 1,4-dioxane | Heated to 110 °C | Aggregation |
| Acetone | Heated to 70 °C | Insoluble |
| EtOAc | Heated to 90 °C | Insoluble |
| Toluene | Heated to 110 °C | Insoluble |
| DMF | Heated to 120 °C | Dissolved |
| DMSO | Heated to 120 °C | Dissolved |

**Gelation of (*S*)-9$_2$:Ca**

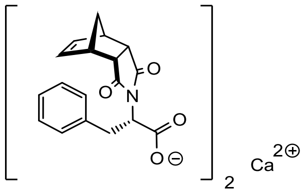


(*S*)-9$_2$:Ca

**Table S3.9.** Solvent gelation using **(*S*)-9$_2$:Ca** at 4 mg/200 µL (2 Wt%)

| Solvent | Experiment | Observation |
|---|---|---|
| MeOH | Heated to 80 °C | Insoluble |
| EtOH | Heated to 90 °C | Insoluble |
| *i*-PrOH | Heated to 90 °C | Insoluble |
| *n*-BuOH | Heated to 120 °C | Insoluble |
| $CHCl_3$ | Heated to 70 °C | Insoluble |
| $CH_2Cl_2$ | Heated to 70 °C | Insoluble |
| THF | Heated to 80 °C | Insoluble |
| $CH_3CN$ | Heated to 110 °C | Insoluble |
| 1,4-dioxane | Heated to 110 °C | Insoluble |
| Acetone | Heated to 70 °C | Insoluble |
| EtOAc | Heated to 90 °C | Insoluble |
| Toluene | Heated to 110 °C | Insoluble |
| DMF | Heated to 120 °C | Dissolved |
| DMSO | Heated to 120 °C | Dissolved |

**Gelation of (*S*)-9:TMA**

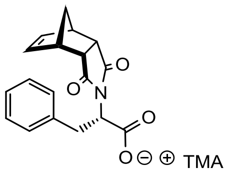


**(*S*)-9:TMA**

**Table S3.10.** Solvent gelation using **(*S*)-9:TMA** at 4 mg/200 μL (2 Wt%)

| Solvent | Experiment | Observation |
|---|---|---|
| MeOH | Heated to 80 °C | Dissolved |
| EtOH | Heated to 90 °C | Dissolved |
| *i*-PrOH | Heated to 90 °C | Dissolved |
| *n*-BuOH | Heated to 120 °C | Dissolved |
| $CHCl_3$ | Heated to 70 °C | Dissolved |
| $CH_2Cl_2$ | Heated to 70 °C | Dissolved |
| THF | Heated to 80 °C | Dissolved |
| $CH_3CN$ | Heated to 110 °C | Dissolved |
| 1,4-dioxane | Heated to 110 °C | Dissolved |
| Acetone | Heated to 70 °C | Dissolved |
| EtOAc | Heated to 90 °C | Dissolved |
| Toluene | Heated to 110 °C | Dissolved |
| DMF | Heated to 120 °C | Dissolved |
| DMSO | Heated to 120 °C | Dissolved |

**Gelation of (*S*)-9:TEA**

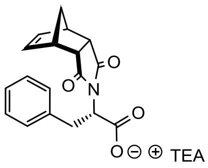


**(*S*)-9:TEA**

**Table S3.11.** Solvent gelation using **(*S*)-9:TEA** at 8 mg/400 μL (2 Wt%)

| Solvent | Experiment | Observation |
|---|---|---|
| MeOH | Heated to 80 °C | Dissolved |
| EtOH | Heated to 90 °C | Dissolved |
| *i*-PrOH | Heated to 90 °C | Dissolved |
| *n*-BuOH | Heated to 120 °C | Dissolved |
| $CHCl_3$ | Heated to 70 °C | Dissolved |
| $CH_2Cl_2$ | Heated to 70 °C | Dissolved |
| THF | Heated to 80 °C | Dissolved, slow formation of crystals |
| $CH_3CN$ | Heated to 110 °C | Dissolved |
| 1,4-dioxane | Heated to 110 °C | Dissolved |
| Acetone | Heated to 70 °C | Dissolved |
| EtOAc | Heated to 90 °C | Dissolved, slow formation of needle-like crystals |
| Toluene | Heated to 110 °C | Dissolved |
| DMF | Heated to 120 °C | Dissolved |
| DMSO | Heated to 120 °C | Dissolved |

**Gelation of (*S*)-9:TBA**

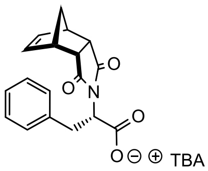


**(*S*)-9:TBA**

**Table S3.12.** Solvent gelation using **(*S*)-9:TBA** at 4 mg/200 μL (2 Wt%)

| Solvent | Experiment | Observation |
|---|---|---|
| MeOH | Heated to 80 °C | Dissolved |
| EtOH | Heated to 90 °C | Dissolved |
| *i*-PrOH | Heated to 90 °C | Dissolved |
| *n*-BuOH | Heated to 120 °C | Dissolved |
| $CHCl_3$ | Heated to 70 °C | Dissolved |
| $CH_2Cl_2$ | Heated to 70 °C | Dissolved |
| THF | Heated to 80 °C | Dissolved |
| $CH_3CN$ | Heated to 110 °C | Dissolved |
| 1,4-dioxane | Heated to 110 °C | Dissolved |
| Acetone | Heated to 70 °C | Dissolved |
| EtOAc | Heated to 90 °C | Dissolved |
| Toluene | Heated to 110 °C | Dissolved |
| DMF | Heated to 120 °C | Dissolved |
| DMSO | Heated to 120 °C | Dissolved |

**S3.2. Gelator solution**

A concentrated solution of compound was made by dissolving **(*S*)-9-Na** (50 mg) in $H_2O$ (50 μL).

A 5 μL aliquot of the above solution was added to different organic solvent (1.0 mL). *i.e*. 5 mg/5 μL($H_2O$) + organic solvent = 0.5 wt%.

Gels were formed upon sonication. For THF, no sonication was required to elicit complete gelation.

**Table S3.13.** Solvent gelation using **(*S*)-9:Na** (0.5 wt% aqueous solution added to 1 mL of solvent)

| Solvent | Experiment | Result |
|---|---|---|
| MeOH | Sonicated | Miscible |
| EtOH | Sonicated | Miscible |
| *i*-PrOH | Sonicated | **Gel** |
| *n*-BuOH | Sonicated | **Gel** |
| $CHCl_3$ | Sonicated | Immiscible |
| $CH_2Cl_2$ | Sonicated | Immiscible |
| THF | Gel formed before sonication | **Gel** |
| $CH_3CN$ | Sonicated | Immiscible |
| 1,4-dioxane | Sonicated | **Gel** |
| Acetone | Sonicated | **Gel** |
| EtOAc | Sonicated | Immiscible |

## S4. SEM images

### Instrumentation

Gel samples were prepared by placing a small amount of gels (~10 – 15 mL by vol.) on silicon wafers and making a thin film by smooth smearing. The samples were dried initially in air for 12 hours followed by drying in high vacuum for 2 - 3 hours. Before recording the images, the samples were gold coated using sputter. These samples were imaged in Carl Zeiss ULTRA Plus, field emission scanning electron microscope (FE-SEM) using beam current of 5 - 10 kV with an SE2 or InLens detector.

### Preparation samples for SEM analysis

A small scoop of gel (approx. 10 μL by vol) was placed on a Silica wafer and spread into a thin film. This was subsequently dried in air overnight then under vacuum for a further 2 hrs. These samples were then coated with Au using sputter coating.

### Potassium salt (*S*)-9:K

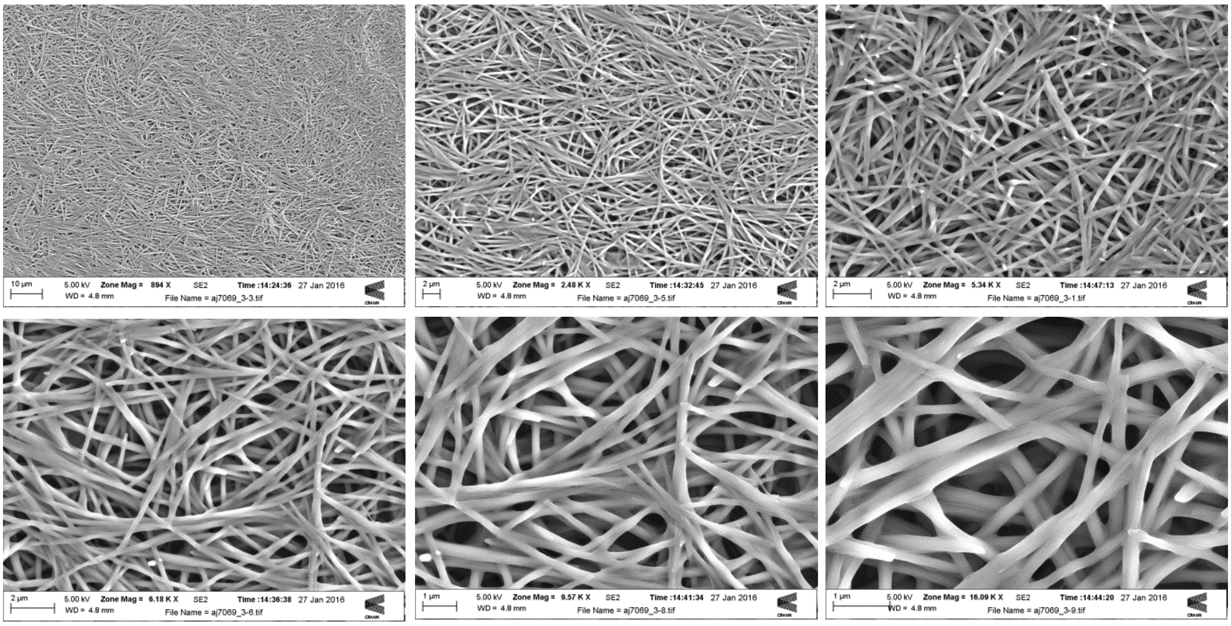

***Figure S4.1***. *SEM Images of* ***(S)-9:K*** *(2 wt%) i-PrOH gel.*

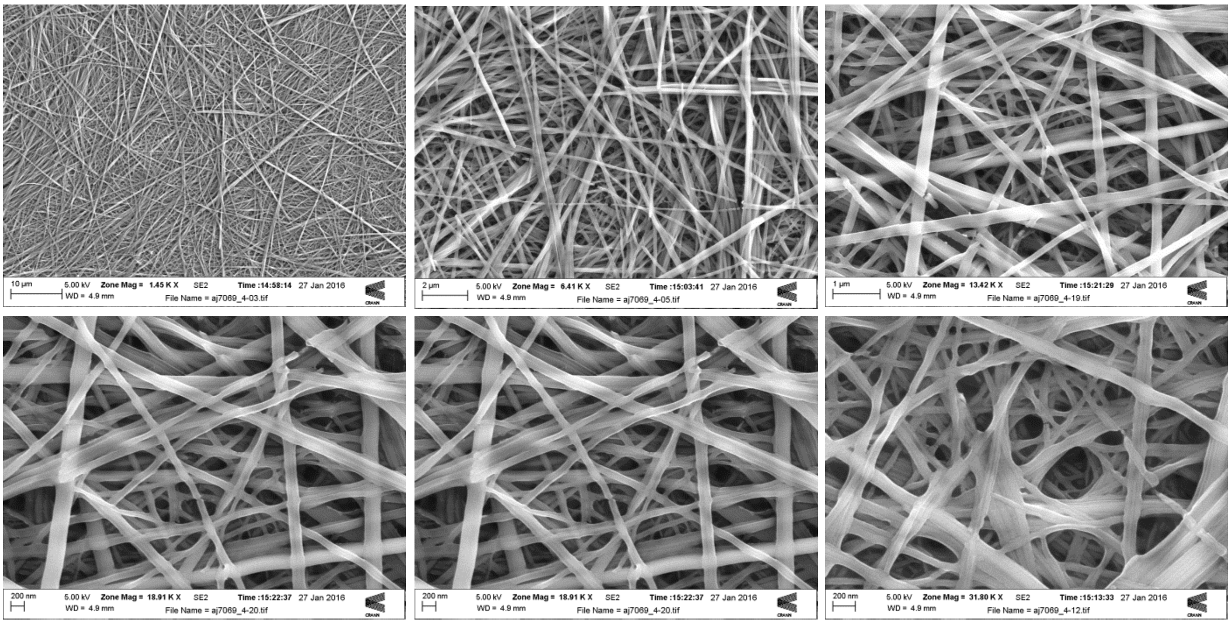

***Figure S4.2.*** *SEM images of* ***(S)-9:K*** *(2 wt%) n-BuOH gel.*

**Sodium salt (*S*)-9:Na**

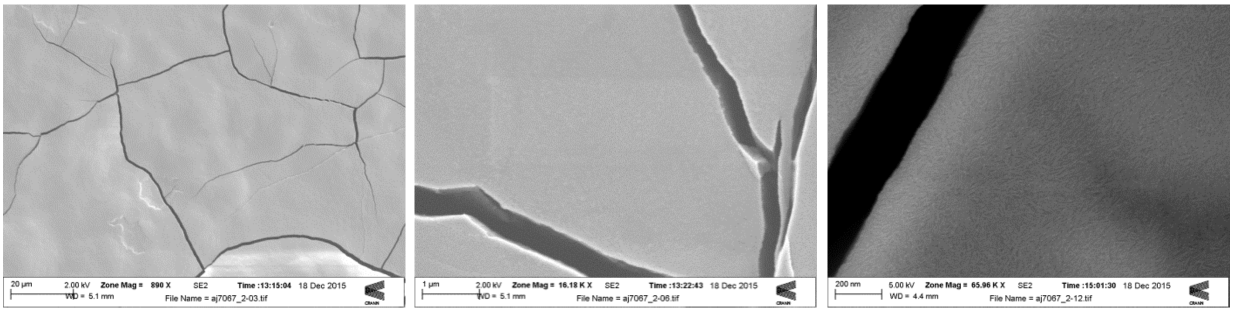

***Figure S4.3.*** *SEM images of* ***(S)-9:Na*** *(2 wt%) EtOH gel.*

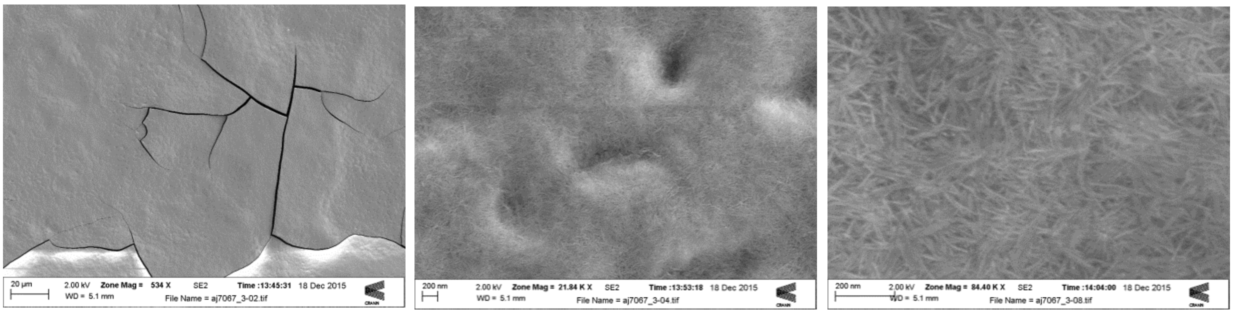

***Figure S4.4***. *SEM images of* ***(S)-9:Na*** *(2 wt%) i-PrOH gel.*

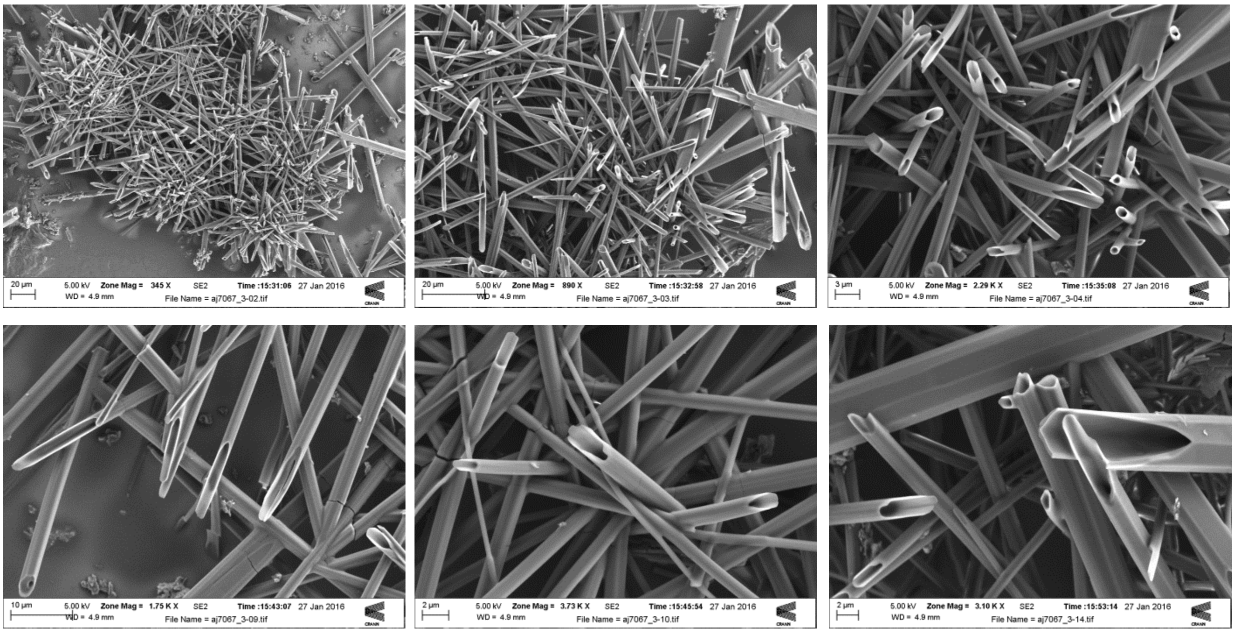

***Figure S4.5**. SEM images of **(S)-9:Na** (1 wt%) i-PrOH gel.*

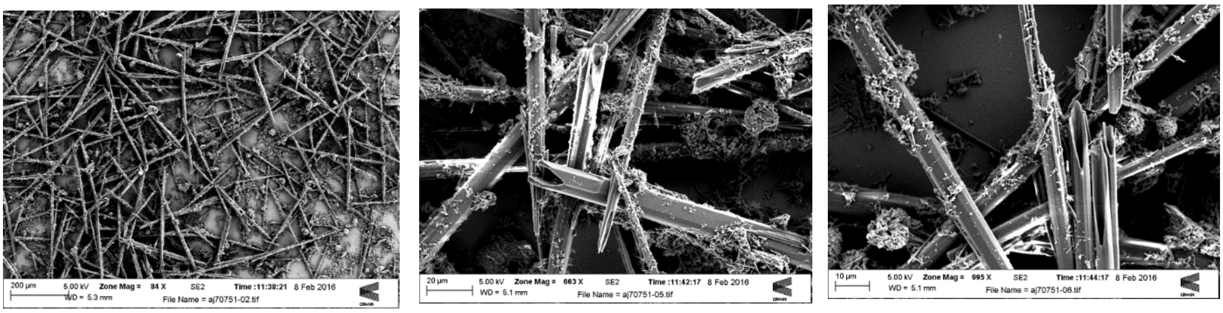

***Figure S4.6**. SEM images of **(S)-9:Na** (0.5 wt%) i-PrOH gel (gelator added as a 3 M aqueous solution).*

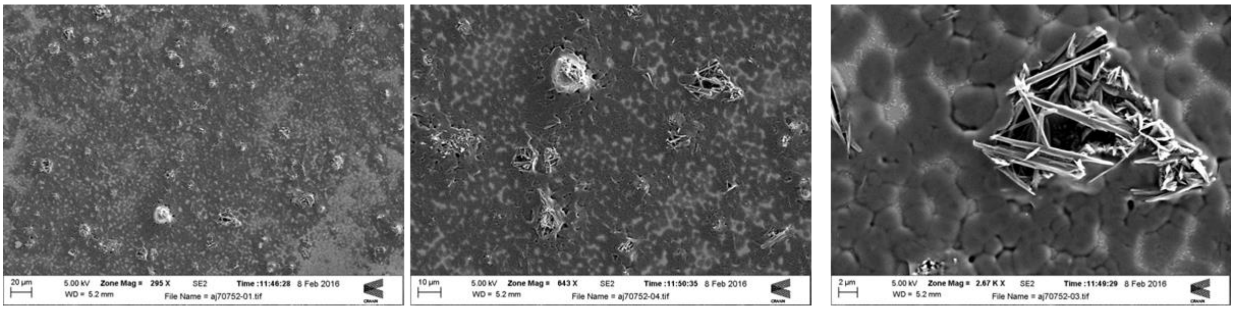

***Figure S4.7**. SEM images of **(S)-9:Na** (0.5 wt%) tetrahydrofuran gel (gelator added as a 3 M aqueous solution).*

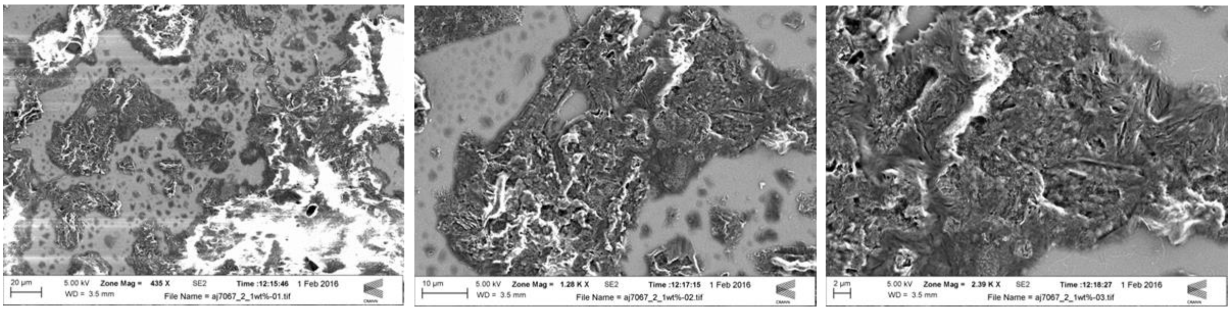


***Figure S4.8***. *SEM images of* ***(S)-9:Na*** *(0.5 wt%) 1,4-Dioxane gel (gelator added as a 3 M aqueous solution)*

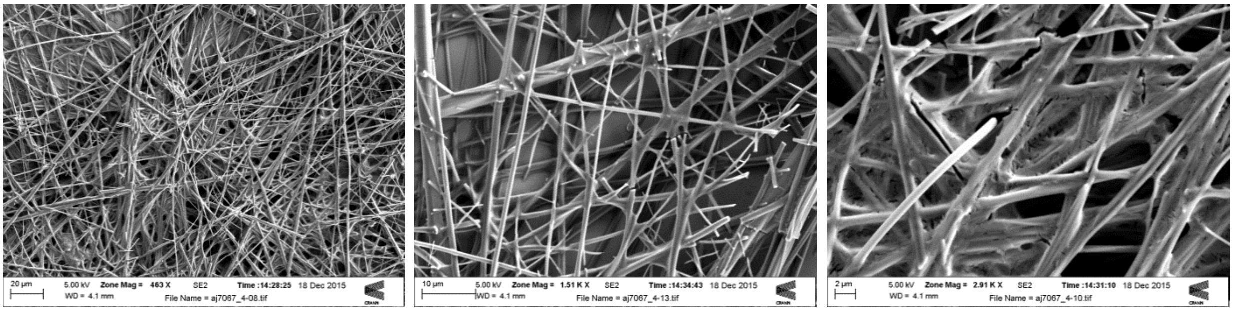


***Figure S4.9***. *SEM images of* ***(S)-9:Na*** *(2 wt%) n-BuOH gel.*

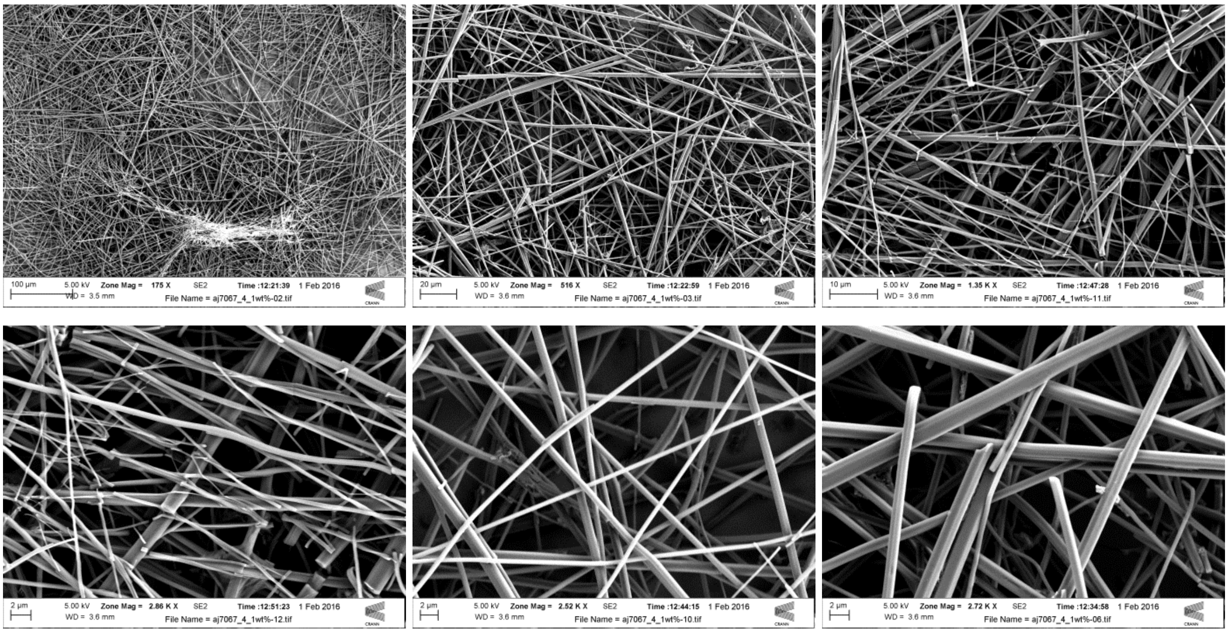


***Figure S4.10***. *SEM images of* ***(S)-9:Na*** *(1 wt%) n-BuOH gel.*

**Lithium salt (*S*)-9:Li**

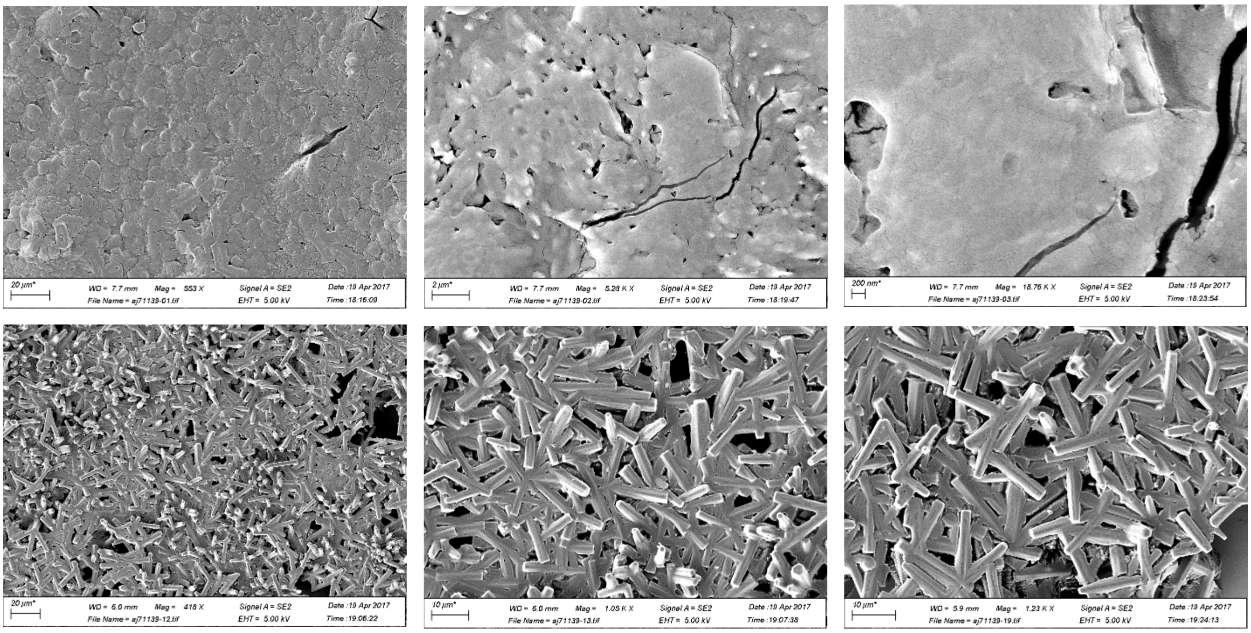

***Figure S4.11**. SEM images of **(S)-9:Li** (2 wt%) 1,4-Dioxane <u>partial</u> gel.* TOP: When a thin SEM sample was prepared, a film was formed; BOTTOM: When a thick SEM sample was used, micron sized crystals were observed.

## S5. DSC calorimetry

### Instrumentation

All DSC measurements were obtained using a TA Instruments DSC Q200 instrument using a Tzero aluminum hermetic pan and lid. A small sample of the gel (approx. 8-10 mg) was placed into the pan and the lid was then crimped on. A small hole was punctured into the lid, carefully avoiding disturbing the sample, and the pan was then placed in the sample port. An air flow rate of 50.0 mL/min was employed. The samples were allowed to equilibrate at 20 °C before being heated at 1 °C/min.

**Table S5.1.** Gel-sol transition points of formed organogels at MGC obtained from DSC measurements.

| Gel | Gel-sol transition point (°C)[a] |
|---|---|
| **9:Na** – Ethanol | 47 |
| **9:Na** – Isopropanol | 55 |
| **9:Na** – *n*-Butanol | 89 |
| **9:Na** – 1,4-Dioxane | 73 |
| **9:Na** – THF | 27 |
| **9:Na** – Acetone | 32 |
| **9:Na** – Chloroform | 27 |
| **9:K** – Isopropanol | 58 |
| **9:K** – *n*-Butanol | 83 |

### Potassium salt (*S*)-9:K

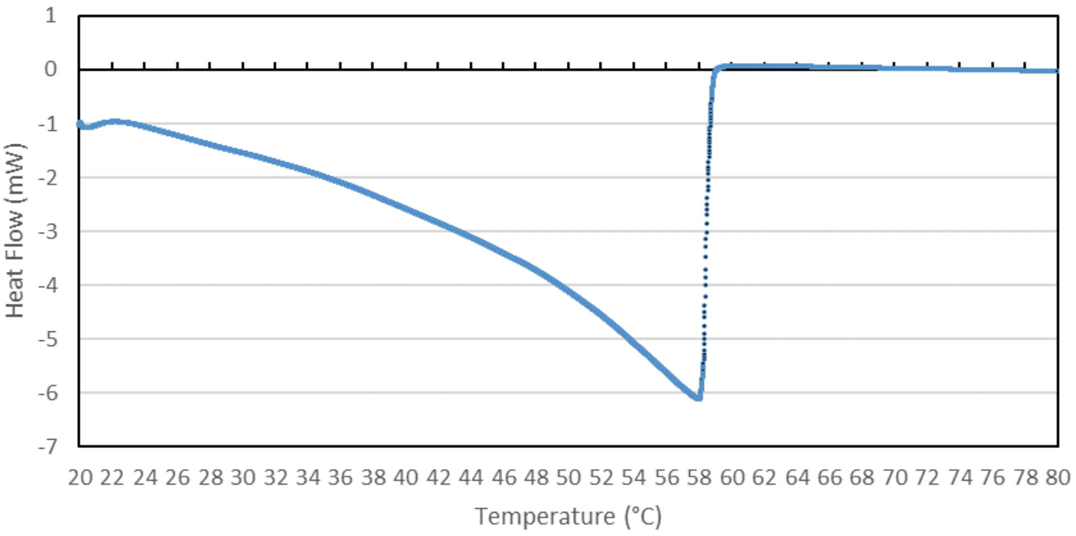


***Figure S5.1**: DSC thermograph of a gel formed by 1 wt% **(S)-9:K** in i-PrOH*

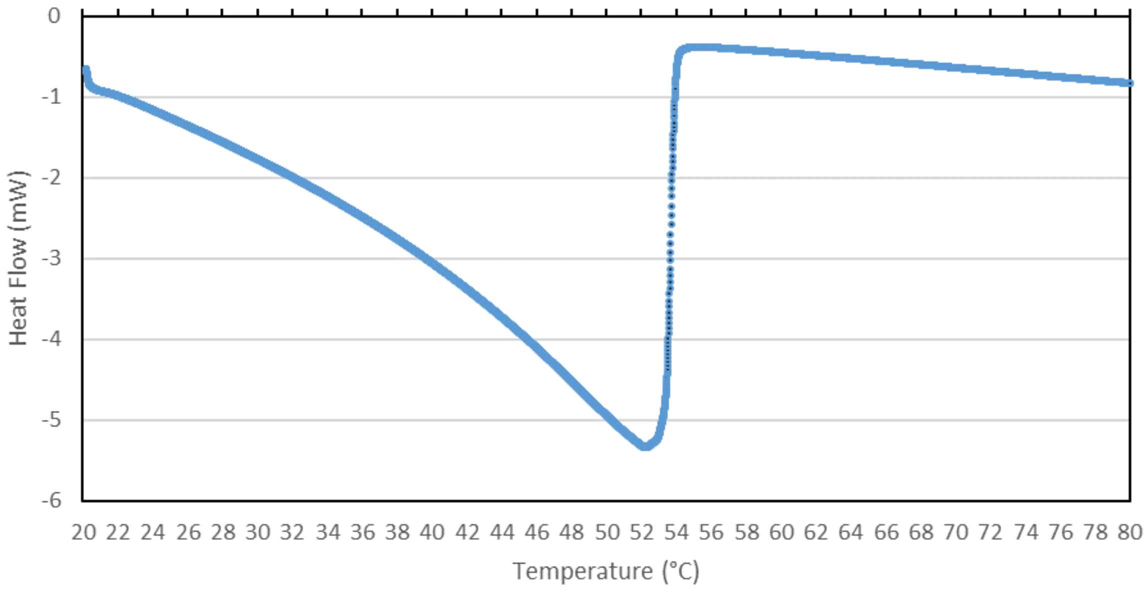


***Figure S5.2***: *DSC thermograph of a gel formed by 1 wt%* ***(S)-9:K*** *in t-BuOH*

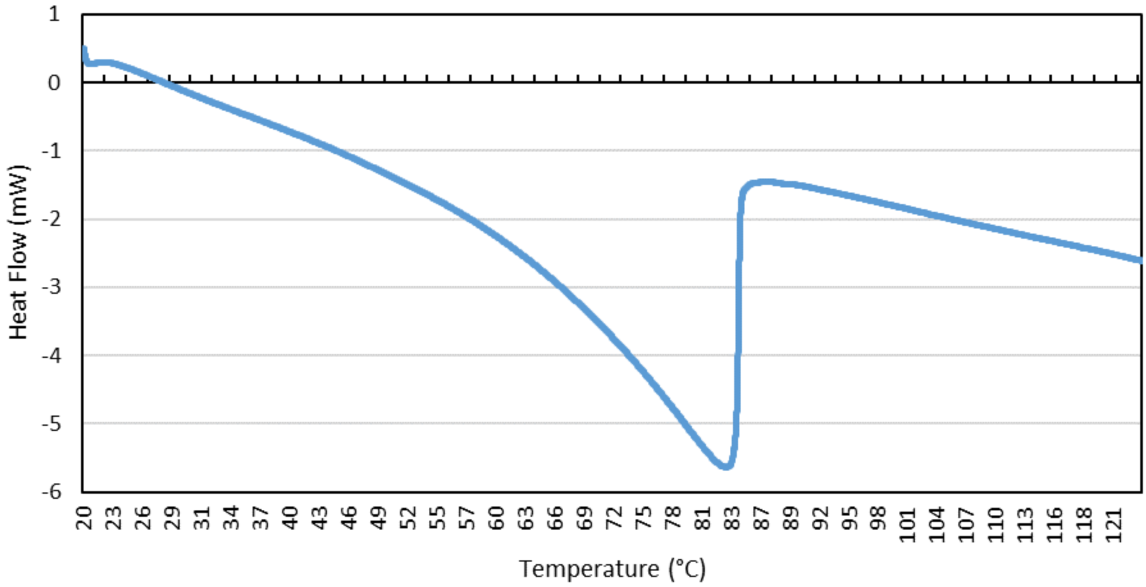


***Figure S5.3***: *DSC thermograph of a gel formed by 1 wt%* ***(S)-9:K*** *in n-BuOH*

**Sodium salt (*S*)-9:Na**

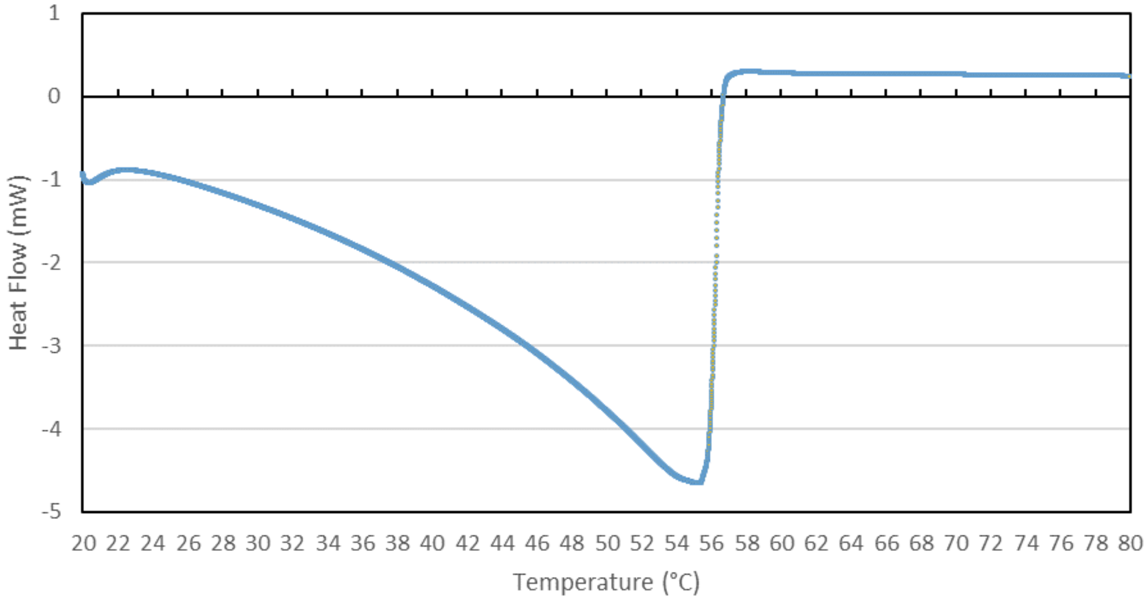


***Figure S5.4**: DSC thermograph of a gel formed by 1 wt% **(S)-9:Na** in i-PrOH*

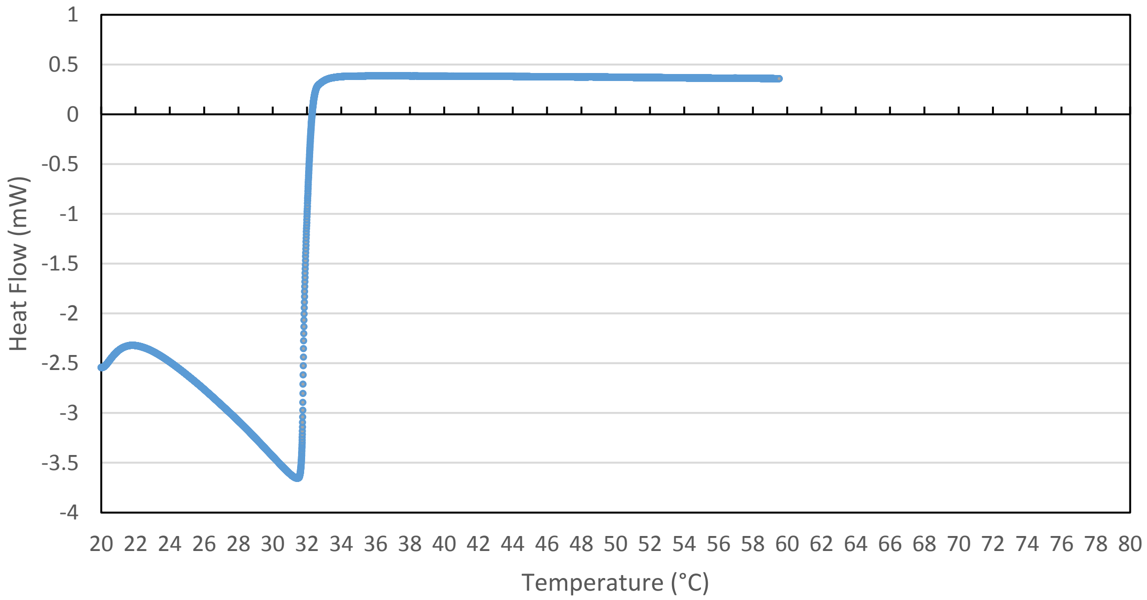


***Figure S5.5**: DSC thermograph of a gel formed by 1 wt% **(S)-9:Na** in acetone*

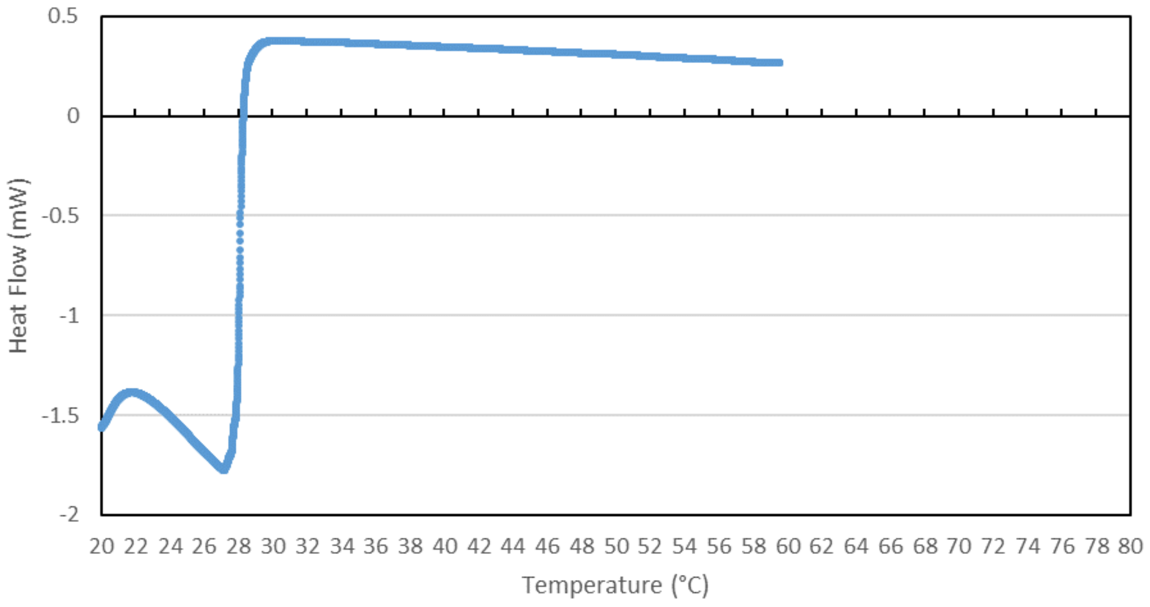


***Figure S5.6**: DSC thermograph of a gel formed by 1 wt% **(S)-9:Na** in $CH_3Cl$*

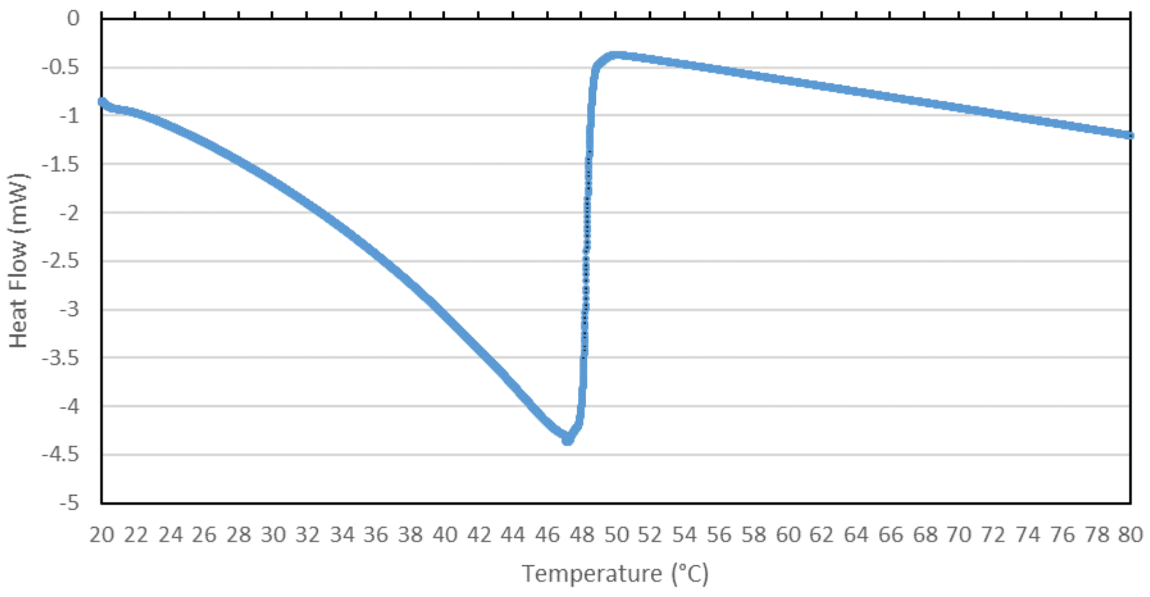


***Figure S5.7**: DSC thermograph of a gel formed by 2 wt% **(S)-9:Na** in EtOH*

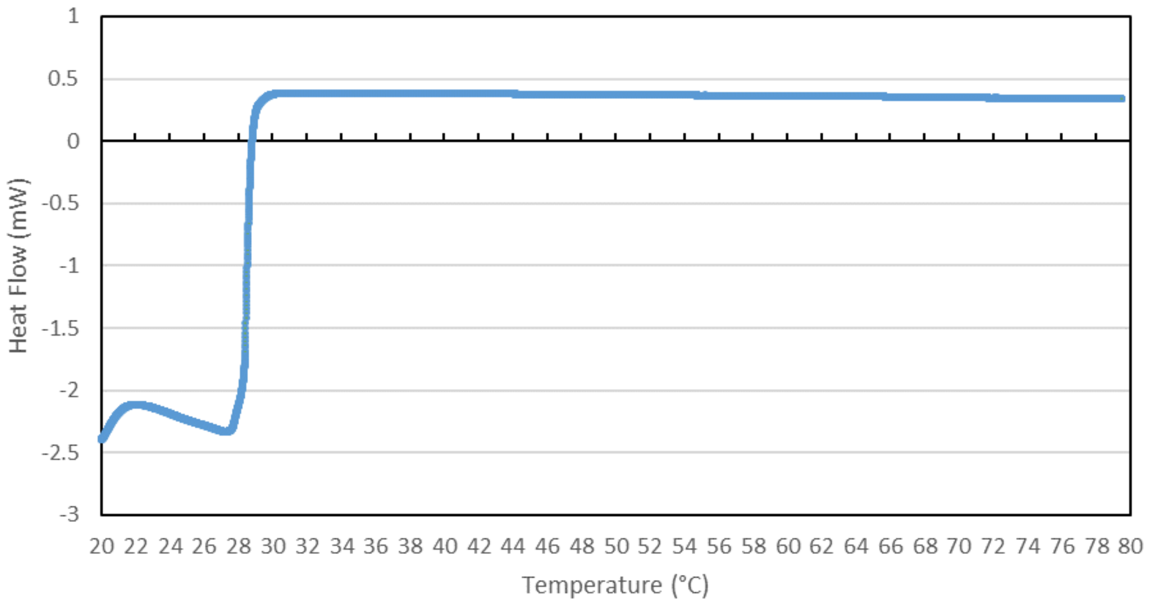


***Figure S5.8**: DSC thermograph of a gel formed by 1 wt% **(S)-9:Na** in tetrahydrofuran*

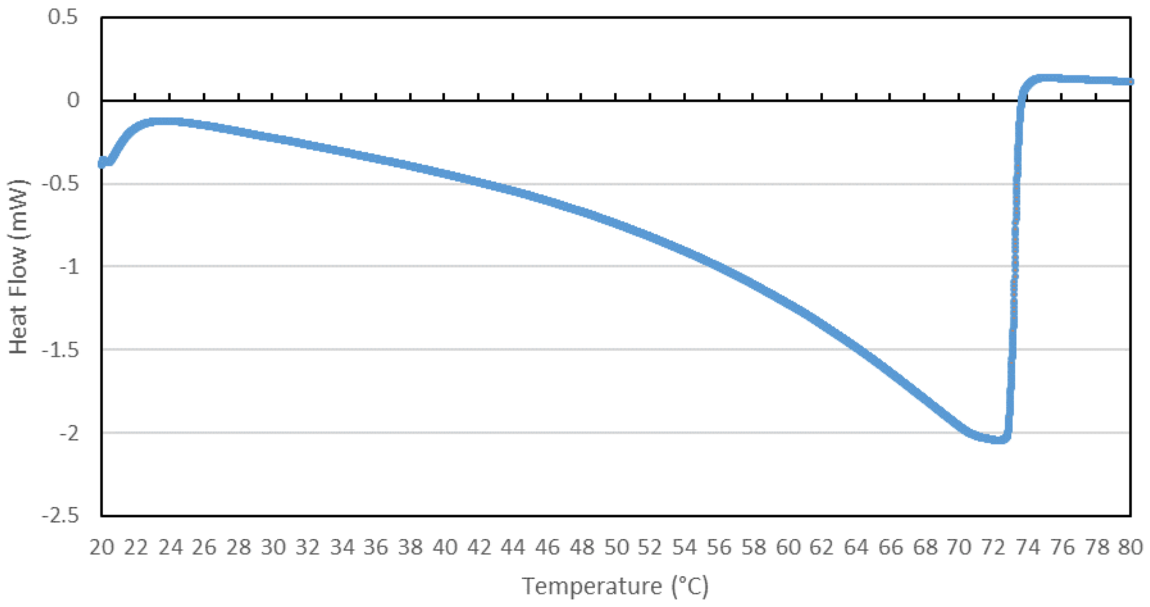


***Figure S5.9**: DSC thermograph of a gel formed by 1 wt% **(S)-9:Na** in 1,4-Dioxane*

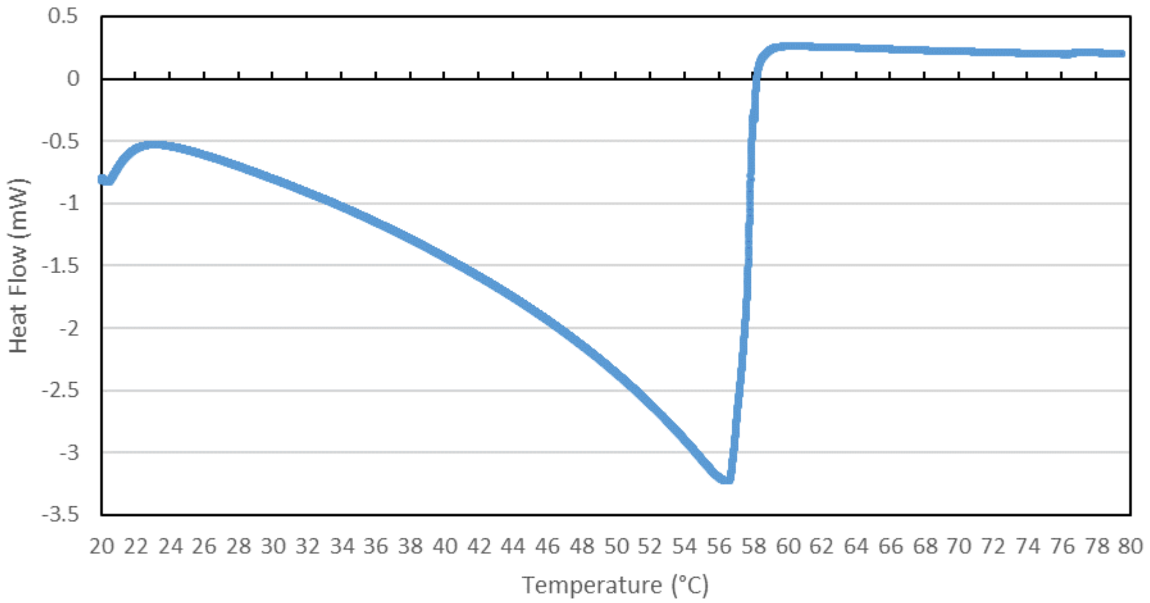


***Figure S5.10***: *DSC thermograph of a gel formed by 1 wt%* ***(S)-9:Na*** *in t-BuOH*

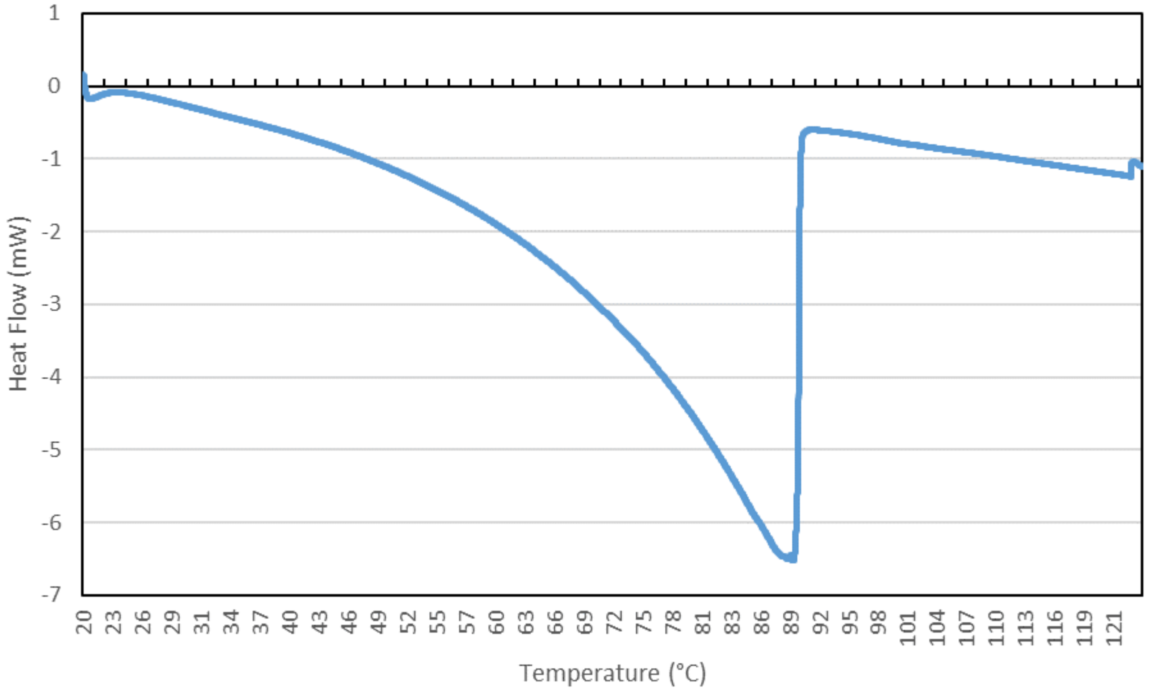


***Figure S5.11***: *DSC thermograph of a gel formed by 1 wt%* ***(S)-9:Na*** *in n-BuOH*

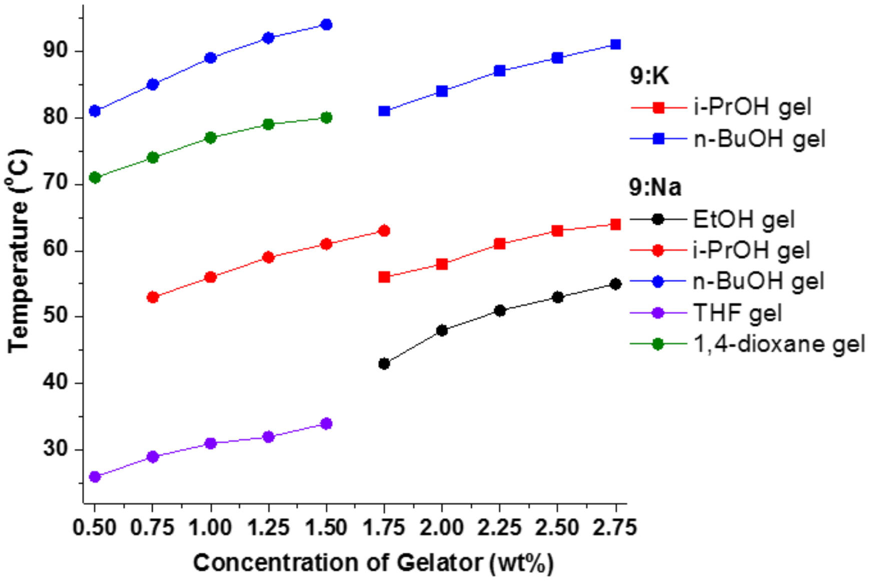


***Figure S5.12**: Variation of $T_{gel}$ vs concentration of gelator in different solvents.*

Thermal stability ($T_{gel}$) of **9:K** and **9:Na** gels prepared by increasing the gelator concentrations was measured by ball drop method. $T_{gel}$ values of gels increased with increasing concentration of gelator. Variation of Tgel vs concentration is shown in **Figure S5.12**. **9:K** and **9:Na** gels in EtOH, i-PrOH, n-BuOH, were formed by heating solid powder in organic solvents and cooling to room temperature. Whereas, **9:Na** gels in THF and 1,4-dioxane were formed by adding aqueous solution (c = 100%) of gelator to organic solvents.

## S6. Rheology

### Instrumentation

Rheological measurements were conducted on a Discovery HR-3 hybrid rheometer with a 40 mm 1.995° cone plate geometry at 25 °C. The set gap was approximately 0.5 mm for all samples. Steady shear measurements (flow sweep) were carried out in the shear rate range of 0.1–5000 $s^{-1}$ which is the upper limit of the instrument.

### Potassium salt (*S*)-9:K

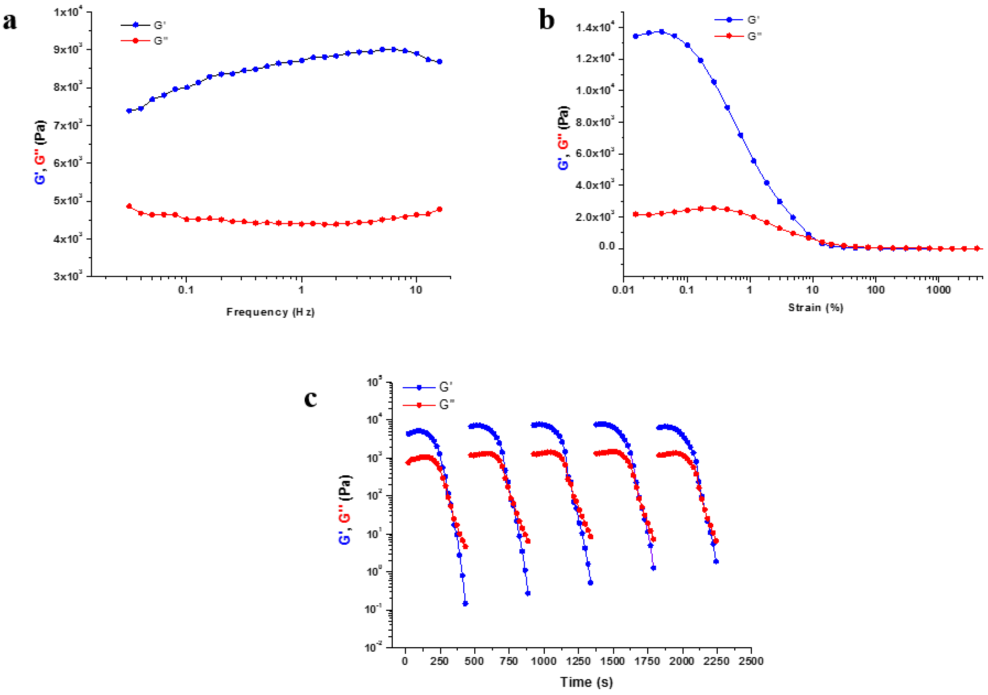


**Figure S6.1 (S)-9:K** 4 mg (2 Wt%) in i-PrOH A) Frequency sweep B) Strain sweep and C) Recovery test.

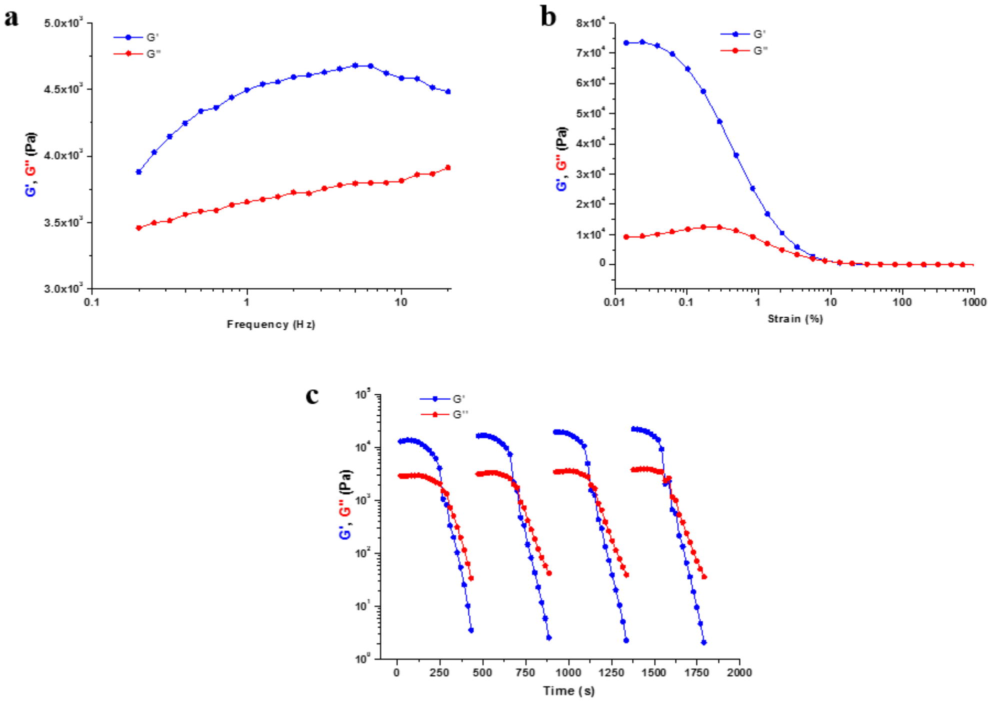


**Figure S6.2**. **(S)-9:K** at 4 mg (2 Wt%) in t-BuOH A) Frequency sweep B) Strain sweep and C) recovery test.

## Sodium Salt (*S*)-9:Na

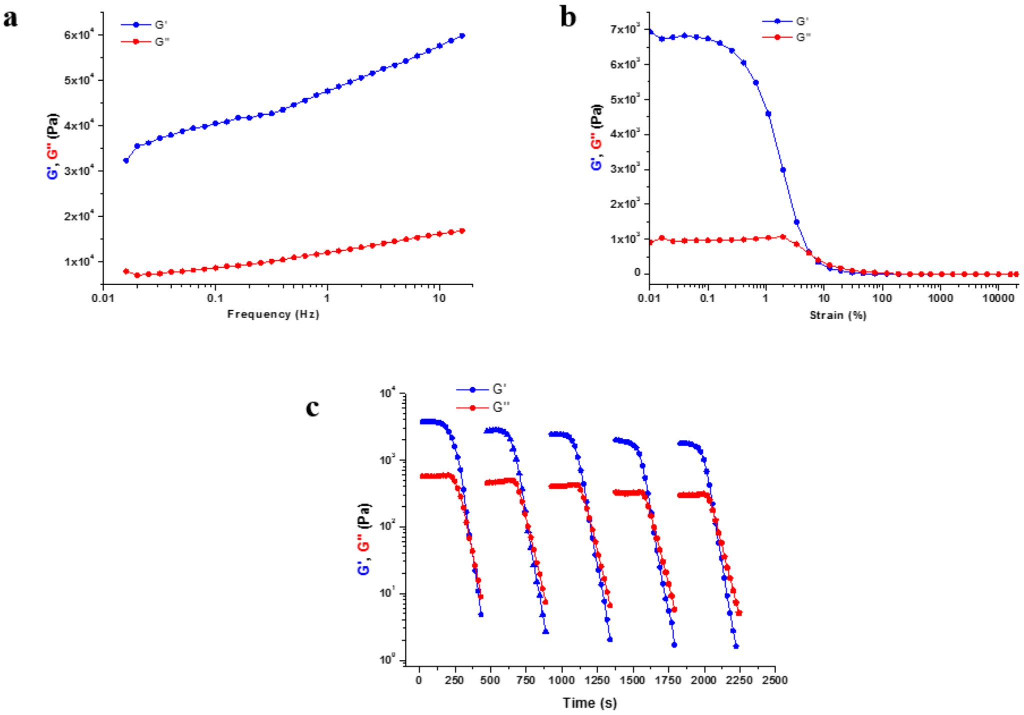


**Figure S6.3**: **(*S*)-9:Na** at 4 mg (2 Wt%) in EtOH A) Frequency sweep B) Strain sweep and C) recovery test.

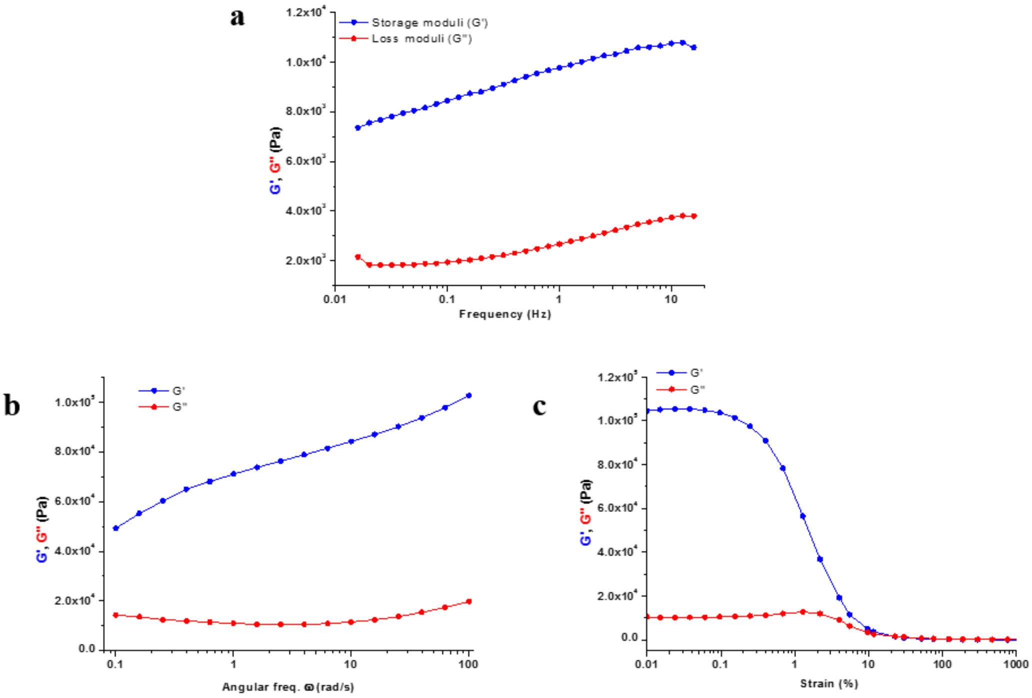


***Figure S6.4***: **(*S*)-9:Na** in *i*-PrOH *A) Frequency sweep rheological studies for the i-PrOH gel (1 wt.%) of* ***9:Na****. (B-C) Frequency sweep and Strain sweep rheological studies for the i-PrOH gel (2 wt.%) of* **9:Na**.

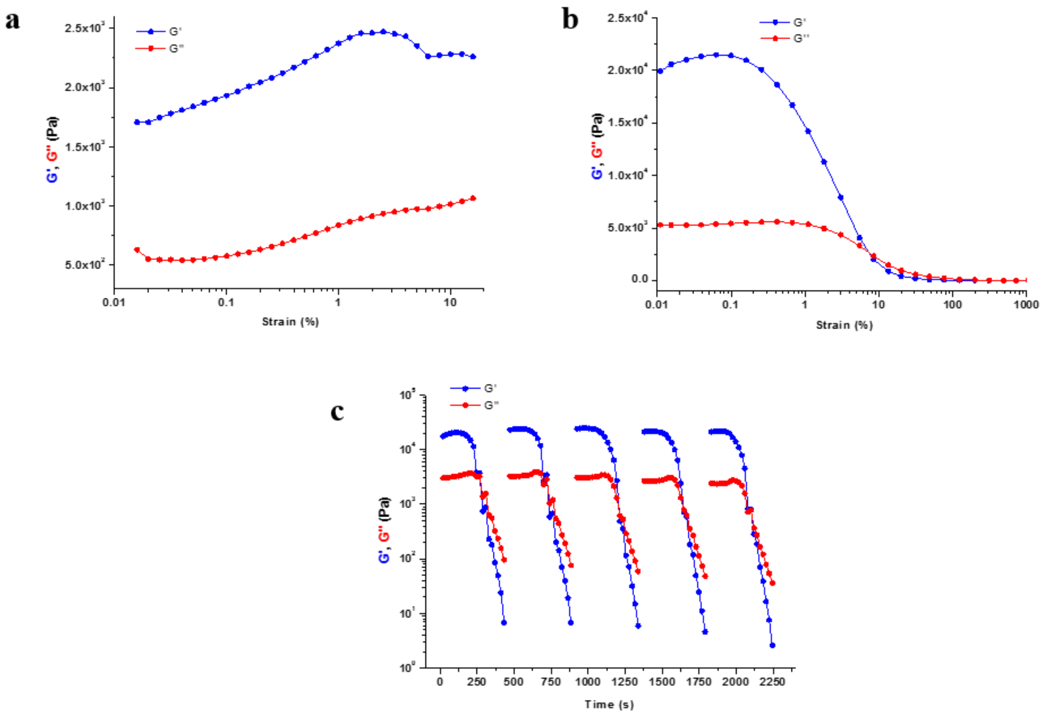


***Figure S6.5*: *(S)-9:Na at 4 mg (2 Wt%) in n-BuOH A) Frequency sweep B) Strain sweep and C) Recovery test.***

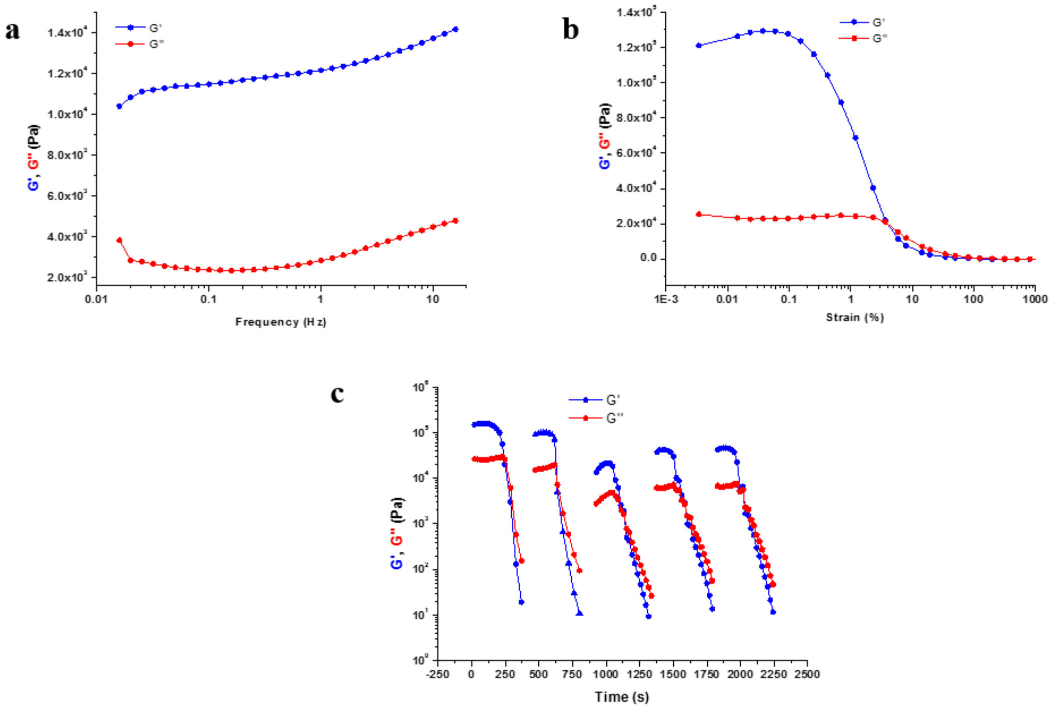


**Figure S6.6**: **(*S*)-9:Na** at 4 mg (2 Wt%) in 1,4-dioxane A) Frequency sweep, B) Strain sweep C) Recovery test.

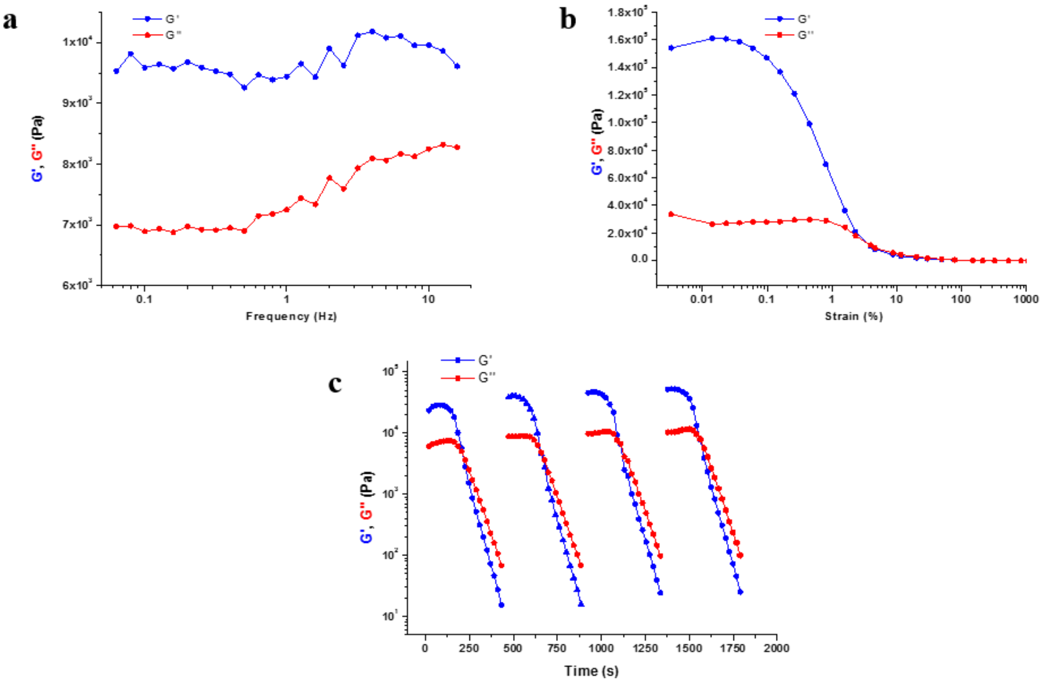


**Figure S6.7**: **(S)-9:Na** at 4 mg (2 Wt%) in $CHCl_3$ A) Frequency sweep, B) Strain sweep and C) Recovery test.

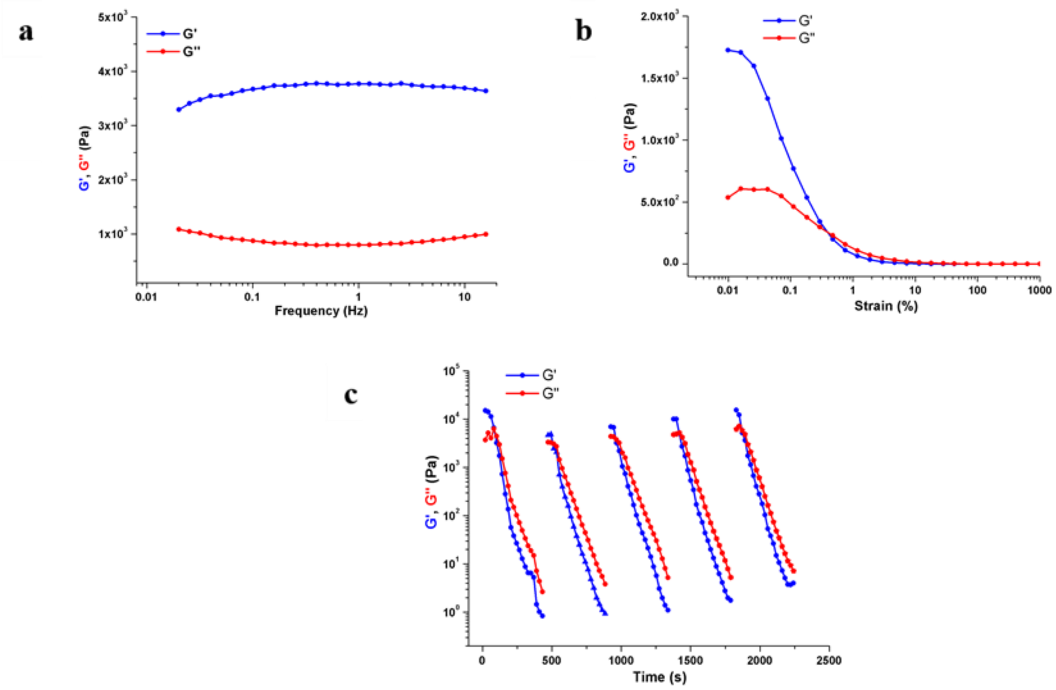


**Figure S6.8**: **(S)-9:Na** at 4 mg (2 Wt%) in THF A) Frequency sweep, B) Strain sweep and C) Recovery test.

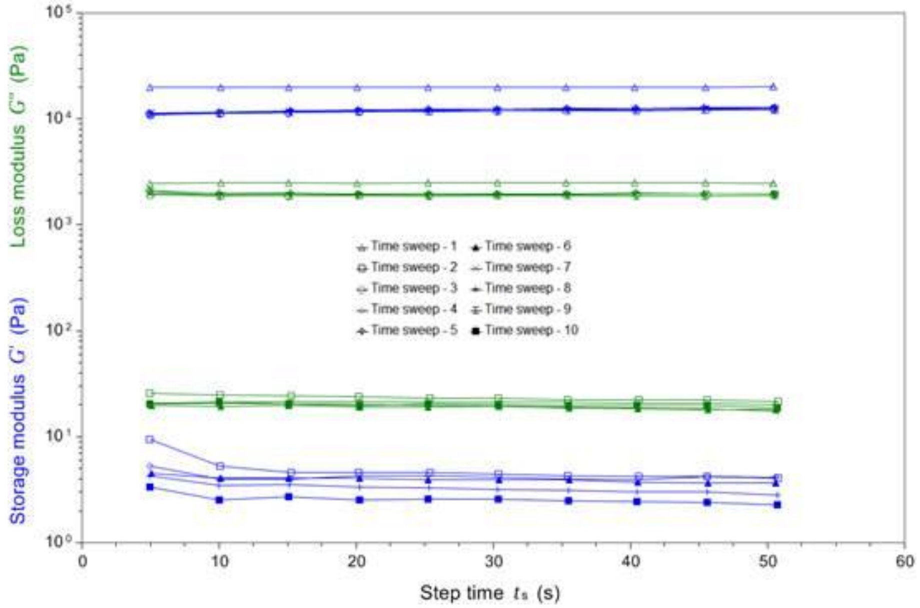


**Figure S6.9**: Gel recovery experiment. A **9:Na** gel sample in i-PrOH (1 Wt%) was tested at low strain (0.01 %) then high strain (100%) to liquefy the gel. This cycle of liquefaction then gelling was repeated a further 4 times to demonstrate both the recovery of the gels and the consistency of the G' values.

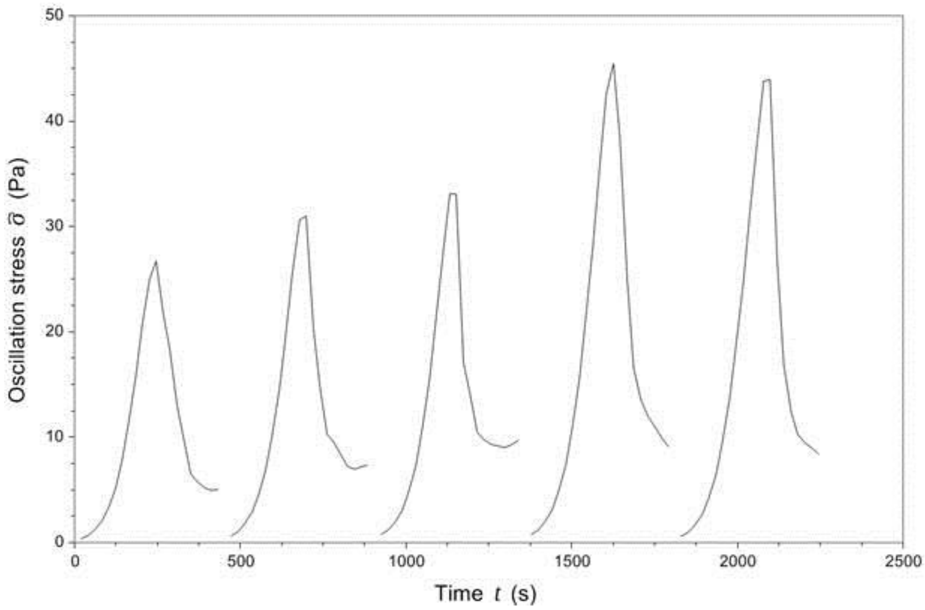


**Figure S6.10**: Examining data for plate slippage. A plot of the oscillation stress over time shows no plate slippage over the course of the recovery experiments performed above. Note that there are no sudden "drops" or "shifts" in the stress measured (these would indicate slippage). The magnitudes and trends in the stress measured are consistent and correspond well to the experimental design as would be expected for reversible gel behaviour.

## S7. Crystallography

### Instrumentation and data refinement

Crystal and refinement parameters are shown below (Table S7.1).

**Table S7.1**. Crystallographic and Refinement Parameters for all datasets

| Identification code | (R)-9:Na | (S)-9:Na | (S)-9:TEA |
|---|---|---|---|
| Empirical formula | $C_{18}H_{17}NNaO_5$ | $C_{18}H_{17}NNaO_5$ | $C_{26}H_{40}N_2O_6$ |
| Formula weight | 350.31 | 350.31 | 476.6 |
| Temperature/K | 130.01(10) | 130.01(10) | 100 |
| Crystal system | hexagonal | hexagonal | triclinic |
| Space group | $P6_3$ | $P6_3$ | $P1$ |
| a/Å | 20.8091(10) | 20.7637(3) | 8.8976(3) |
| b/Å | 20.8091(10) | 20.7637(3) | 9.7399(3) |
| c/Å | 6.5883(4) | 6.59150(10) | 15.4155(5) |
| α/° | 90 | 90 | 93.9720(10) |
| β/° | 90 | 90 | 99.6020(10) |
| γ/° | 120 | 120 | 107.2150(10) |
| Volume/Å$^3$ | 2470.6(3) | 2461.07(8) | 1248.18(7) |
| Z | 6 | 6 | 2 |
| $\rho_{calc}$g/cm$^3$ | 1.413 | 1.418 | 1.268 |
| μ/mm$^{-1}$ | 1.084 | 1.088 | 0.726 |
| F(000) | 1098 | 1098 | 516 |
| Crystal size/mm$^3$ | 0.457 × 0.060 × 0.030 | 0.432 × 0.150 × 0.092 | 0.31 × 0.17 × 0.14 |
| Radiation | Cu Kα (λ = 1.5418) | Cu Kα (λ = 1.5418) | Cu Kα (λ = 1.5418) |
| 2Θ range for data collection/° | 8.498 to 134.976 | 8.518 to 154.252 | 5.862 to 136.592 |
| Index ranges | -24 ≤ h ≤ 8, -20 ≤ k ≤ 24, -7 ≤ l ≤ 7 | -18 ≤ h ≤ 26, -22 ≤ k ≤ 26, -5 ≤ l ≤ 8 | -10 ≤ h ≤ 10, -11 ≤ k ≤ 11, -18 ≤ l ≤ 18 |
| Reflections collected | 10790 | 9217 | 33371 |
| Independent reflections | 2849 [$R_{int}$ = 0.0503, $R_{sigma}$ = 0.0495] | 2844 [$R_{int}$ = 0.0245, $R_{sigma}$ = 0.0259] | 9072 [$R_{int}$ = 0.0361, $R_{sigma}$ = 0.0292] |
| Observed Reflections [I>=2σ (I)] | 2528 | 2650 | 9004 |
| Data/restraints/parameters | 2849/15/246 | 2844/17/246 | 9072/11/645 |
| Goodness-of-fit on F$^2$ | 1.039 | 1.117 | 1.03 |
| Final R indexes [I>=2σ (I)] | $R_1$ = 0.0726, $wR_2$ = 0.1768 | $R_1$ = 0.0631, $wR_2$ = 0.1502 | $R_1$ = 0.0340, $wR_2$ = 0.0939 |
| Final R indexes [all data] | $R_1$ = 0.0824, $wR_2$ = 0.1849 | $R_1$ = 0.0694, $wR_2$ = 0.1538 | $R_1$ = 0.0342, $wR_2$ = 0.0941 |
| Largest diff. peak/hole / e Å$^{-3}$ | 0.85/-0.63 | 0.78/-0.70 | 0.41/-0.21 |
| Flack parameter | -0.04(10) | 0.0(2) | 0.05(3) |
| CCDC Number | 1826288 | 1826289 | 1826290 |

The data for **9:Na** (*R* and *S* enantiomers) were collected using microfocus Cu Kα radiation (λ = 1.54184 Å) with an Oxford Diffraction SuperNova instrument equipped with an Atlas CCD detector, with sample maintained at a temperature of 130 K. Data reduction was carried out using Crysalis Pro software package, with empirical absorption correction performed using SCALE3 ABSPACK.[8, 9] The data for **9:TEA** were collected using a Bruker APEX-II DUO instrument using microfocus Cu Kα radiation (λ = 1.54184 Å), with the sample maintained at 100 K using a Cobra cryostream. These data were processed using Bruker APEX3 software, with multi-scan absorption correction carried out with SADABS.[10, 11] All datasets were solved using SHELXT, and refined with SHELXL, operated within the OLEX2 software package.[12-15] The functions minimized were $\Sigma w(F^2_o-F^2_c)$, with $w=[\sigma^2(F^2_o)+aP^2+bP]^{-1}$, where $P=[\max(F_o)^2+2F^2_c]/3$. All non-hydrogen atoms were refined with anisotropic displacement parameters. All carbon-bound hydrogen atoms were placed in calculated

positions and refined with a riding model, with isotropic displacement parameters equal to either 1.2 or 1.5 times the isotropic equivalent of their carrier atoms. Where appropriate, hydrogen atoms involved in hydrogen bonding were located from Fourier residuals and refined with loose distance restrains and riding $U_{iso}$ dependencies. In the case of **9:Na**, both enantiomers exhibited crystallographic disorder on the carboxylate groups, which were modelled in separate orientation with the occupancies refined as free variables, with similar treatment applied to the disordered aqua ligands. The crystallographic formulae were amended to include the hydrogen atoms for these groups which could not be explicitly modelled. In the case of **9:TEA**, in order to achieve acceptable Friedel pair coverage, two datasets were collected on the same crystal with the sample manually reoriented between scans. and the data were merged using Bruker XPREP to achieve >98% Friedel pair coverage.[16] Full details are provided in the combined crystallographic information file. In all cases, the absolute configuration of each material was unambiguously confirmed by anomalous dispersion effects.CCDC 1826290.

Single crystals of both the *R* and *S* enantiomers of **9:Na** were prepared by first forming a gel in *i*-PrOH and allowing the solvent to evaporate slowly. Analysis by single crystal X-ray diffraction was performed and the data for each were solved and refined in the hexagonal space group $P6_3$. Both compounds crystallised in their enantiomerically pure forms (Table S7.1) and exhibited identical (inverted) behaviour to one another. The asymmetric unit of **9:Na** contains one molecule of **9**, deprotonated and coordinating to one unique sodium ion (Figure S7.1). One aqua ligand is also present within the structure which exhibits minor crystallographic disorder, the coincidence of which with the crystallographic sixfold axis also prevents unambiguous location of the associated hydrogen atoms in the structural model. The sodium ion adopts an irregular six-coordinate geometry; the carboxylate group of **9** (which also exhibits minor crystallographic disorder) coordinates in a $\mu_3$-κO;κO,O′;κO′ coordination mode, with symmetry equivalents occupying four coordination sites on each sodium ion (Figure 7.1). The remaining sites are occupied by the aqua ligand and coordination through one imide oxygen atom O2 from the **9** anion. Coordination through the imide oxygen atom is relatively unusual, though is more frequently observed for alkali metal systems than for transition metal or lanthanide complexes;[17-23] in this case, the bond length of 2.315(5) Å is intermediate to the other Na-O distances within the structure which fall in the range 2.042(16) – 2.715(5) Å, accounting for crystallographic disorder.

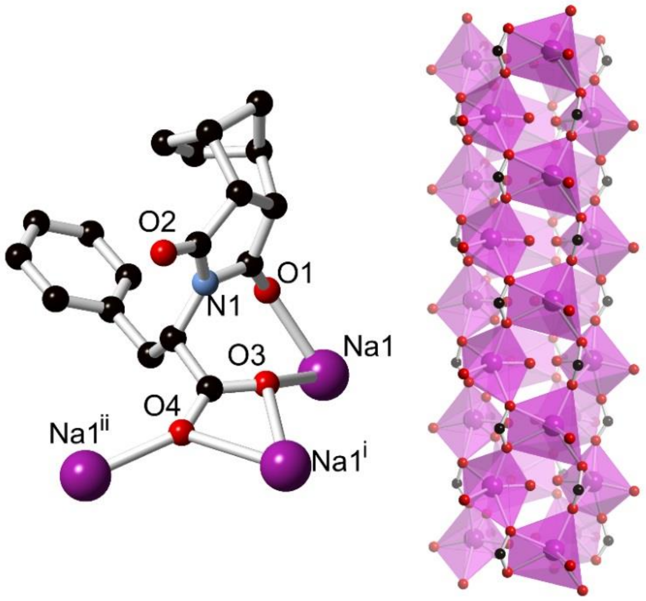


***Figure S7.1** (Left) Partial structure of **9:Na** (S-enantiomer) showing coordination environment of the **9** anion with heteroatom labelling scheme. The carboxylate disorder, hydrogen atoms and aqua ligand are omitted for clarity. Symmetry codes used to generate equivalent atoms: (i) y+x-1, x+1, z-1/2; (ii) +x, +y, z-1.*

Each sodium ion in the structure of **9:Na** connects to three **9** ligands, and the $\mu_3$-bridging behaviour of the **9** ligand gives rise to a one-dimensional polymeric assembly oriented parallel to the crystallographic *c* axis. This takes the form of a one-dimensional tubular assembly with a hydrated sodium-carboxylate core and with the periphery decorated by the phenyl and norbornenyl groups of **9**. The co-incidence of these chains to the $6_3$

symmetry axis imparts a helical twist to these assemblies, with *M* helicity in the **R** enantiomer and *P* helicity in the **S** form. Considering the helices as consisting of alternating phenyl and norbornenyl fragments, which further interact *via* myriad weak C-H···π interactions (*e.g.*, C6-H6···C15, $d_{C\cdots C}$ 3.689(7) Å, C-H···C angle 143.0(4) °), the overall structure is best visualised as a triple-stranded helix (Figure S7.2).

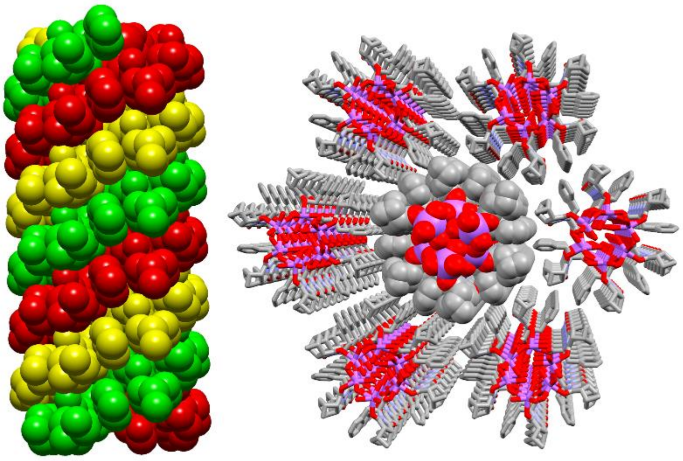

***Figure S7.2.*** *Extended structure of **9:Na**; (Left) a single column of **9:Na** with helical strands coloured separately; (Right) hexagonal rod-packing mode for adjacent stacks in the extended structure of **9:Na**.*

Adjacent stacks in the structure of **9:Na** associate in a hexagonal rod packing fashion with hydrophobic interactions between the outwards facing organic periphery of each chain; no significant voids or crystallographically localised guests were observed in the interstitial spaces, indicating efficient packing of the homochiral helical stacks. This observation may offer some justification as to the lack of any observed crystallisation of the racemate; given the significant undulation in the external surface of the columns and numerous intermolecular contacts occurring in the vicinity of these grooves (clearly visible using Hirshfeld surface mappings, Figure S7.3), co-crystallisation of helices with alternate handedness would be expected to lead to a significant decrease in packing efficiency.

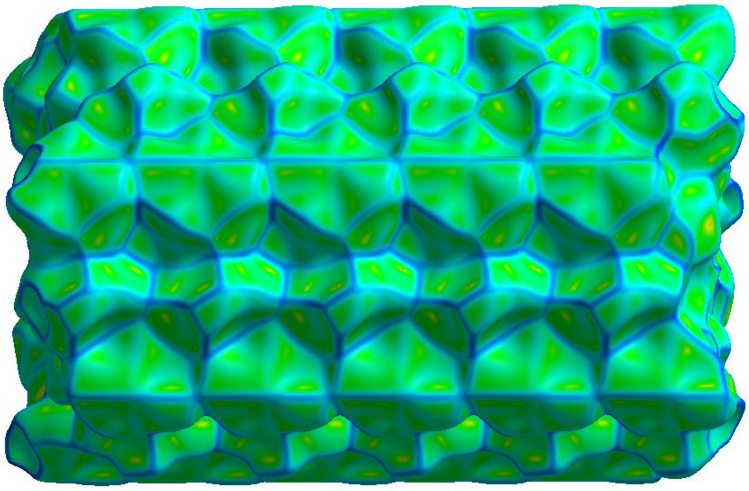

***Figure S7.3.*** *Calculated Hirshfeld surface[16, 24, 25] (isovalue = 0.5) for a segment of the helical stack in **9:Na** rendered with curvedness map (-4.0/+0.4), highlighting the divots in the calculated surface of the stacks caused by close association with neighbouring columns (c axis left-to-right).*

Single crystals of **9:TEA** were prepared by recrystallization from THF and analysed by single crystal X-ray diffraction, and the data were solved and refined in the triclinic space group *P*1. The asymmetric unit contains two deprotonated **9** anions, two tetraethylammonium cations, and four lattice water molecules. Although the

two unique **9** molecules within the unit cell are geometrically similar, the pseudo-inversion symmetry is disrupted by the homochiral nature of the structure, unambiguously confirmed through anomalous dispersion effects (Table S7.1). The **9** anions in **9:TEA** adopt a near-identical conformation to that observed in **9:Na**, with a *gauche* conformation for the phenyl and norbornenyl substituents (given by the torsion angle N1-C10-C12-C13 of 49.2(3) °). This leads to a similar mode o f intramolecular C-H···π interaction to that observed in **9:Na**, with C···C distance of 3.635(4) Å and C-H···C angle 150.3(2)° for the interaction C2-H2···C16. The structure of **9:TEA** is shown in Figure 7.4.

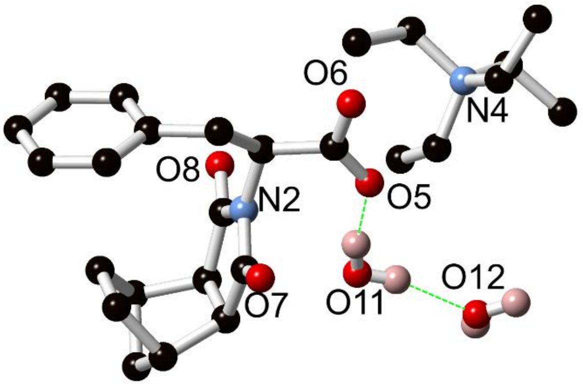


***Figure S7.4.*** *Partial structural representation of* ***9:TEA*** *with heteroatom labelling scheme. For clarity only one of the two crystallographically unique formula units is shown. Hydrogen atoms not participating in hydrogen bonding interactions are omitted for clarity.*

The extended structure of **9.TEA** is dominated by hydrogen bonding interactions between the **9** anion and the lattice water molecules. These interactions take the form of a undulating one-dimensional hydrogen-bonded ribbon consisting of alternating $R_4^4(8)$ planar water tetramers and $R_6^6(16)$ loops involving both water molecules and carboxylate acceptors, oriented parallel to the crystallographic *a* axis. Adjacent ribbons associate *via* face-to-face π-π interactions between phenyl rings (minimum interatomic distance 3.584(4) Å for C16···C31). Cationic {**9**·$2H_2O$} layers are separated by layers of loosely associated tetraethylammonium cations, which contribute numerous weak C-H···O hydrogen bonding interactions within the structure.

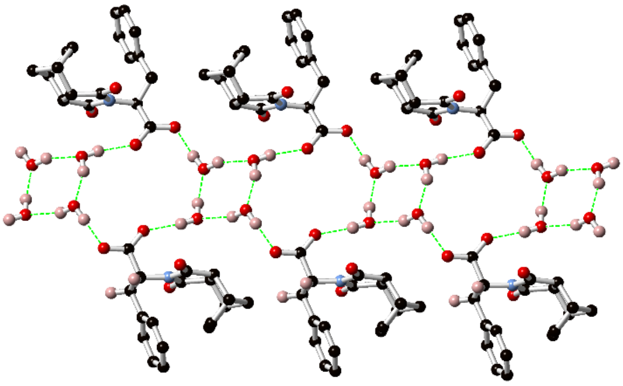

***Figure S7.5.*** *Hydrogen bonding interactions in the structure of* ***9:TEA*** *showing the alternating $R_4^4(8)$ and $R_6^6(16)$ motifs comprising the hydrogen bonded ribbon. Hydrogen atoms not involved in hydrogen bonding, and tetraethylammonium cations are omitted for clarity.*

## 8. References


1. G. Moss, *Pure Appl. Chem.*, 1999, **71**, 513-529.
2. D. L. Pavia, G. M. Lampman, G. S. Kriz and J. A. Vyvyan, *Introduction to spectroscopy*, Cengage Learning, 2008.
3. C. P. Hackenberger, I. Schiffers, J. Runsink and C. Bolm, *The Journal of organic chemistry*, 2004, **69**, 739-743.
4. D. Ranganathan, V. Haridas, S. Kurur, A. Thomas, K. Madhusudanan, R. Nagaraj, A. Kunwar, A. Sarma and I. L. Karle, *J. Am. Chem. Soc.*, 1998, **120**, 8448-8460.
5. H. Koch, J. Kotlan, E. Farkouh and M. Lindner, *Monatshefte für Chemie/Chemical Monthly*, 1971, **102**, 609-621.
6. S. K. Baloch, L. Ma, X.-L. Wang, J. Shi, Y. Zhu, F.-Y. Wu, Y.-J. Pang, G.-H. Lu, J.-L. Qi and X.-M. Wang, *RSC Advances*, 2015, **5**, 31759-31767.
7. E. O. Stejskal and J. E. Tanner, *The journal of chemical physics*, 1965, **42**, 288-292.
8. R. O. D. CrysAlisPro 1.171.38.43, Yarnton, Oxfordshire, England, 2015.
9. R. O. D. SCALE3-ABSPACK, Yarnton, Oxfordshire, England, 2015.
10. B.-A. I. Bruker APEX-3, Madison, WI, 2016.
11. B.-A. I. SADABS, Madison, WI, 2016.
12. D. J. Abdallah and R. G. Weiss, *Adv. Mater.*, 2000, **12**, 1237-1247.
13. O. V. Dolomanov, L. J. Bourhis, R. J. Gildea, J. A. Howard and H. Puschmann, *J. Appl. Crystallogr.*, 2009, **42**, 339-341.
14. G. M. Sheldrick, *Acta Crystallographica Section A: Foundations and Advances*, 2015, **71**, 3-8.
15. G. M. Sheldrick, *Acta Crystallographica Section C: Structural Chemistry*, 2015, **71**, 3-8.
16. J. McKinnon, M. Turner, D. Jayatilaka, M. Spackman, D. Grimwood and S. Wolff, *University of Western Australia*, 2012.
17. J. A. Kitchen, N. Zhang, A. B. Carter, A. J. Fitzpatrick and G. G. Morgan, *J. Coord. Chem.*, 2016, **69**, 2024-2037.
18. J. I. Lovitt, C. S. Hawes, A. D. Lynes, B. Haffner, M. E. Möbius and T. Gunnlaugsson, *Inorganic Chemistry Frontiers*, 2017, **4**, 296-308.
19. S. I. Pascu, C. Naumann, G. Kaiser, A. D. Bond, J. K. Sanders and T. Jarrosson, *Dalton Transactions*, 2007, 3874-3884.
20. D. L. Reger, A. Leitner and M. D. Smith, *Crystal Growth & Design*, 2015, **15**, 5637-5644.
21. D. L. Reger, A. Leitner, M. D. Smith, T. T. Tran and P. S. Halasyamani, *Inorg. Chem.*, 2013, **52**, 10041-10051.
22. L. H. Tong, P. Pengo, W. Clegg, J. P. Lowe, P. R. Raithby, J. K. Sanders and S. I. Pascu, *Dalton Transactions*, 2011, **40**, 10833-10842.
23. C. Xia, S. Wu, Y. Liang, Z. Chen, H. Zou and F. Liang, *Transition Met. Chem.*, 2015, **40**, 839-846.
24. J. J. McKinnon, D. Jayatilaka and M. A. Spackman, *Chem. Commun.*, 2007, 3814-3816.
25. J. J. McKinnon, M. A. Spackman and A. S. Mitchell, *Acta Crystallogr. Sect. B: Struct. Sci.*, 2004, **60**, 627-668.
